# Structural basis for active single and double ring complexes in human mitochondrial Hsp60-Hsp10 chaperonin

**DOI:** 10.1038/s41467-020-15698-8

**Published:** 2020-04-21

**Authors:** Yacob Gomez-Llorente, Fady Jebara, Malay Patra, Radhika Malik, Shahar Nisemblat, Orna Chomsky-Hecht, Avital Parnas, Abdussalam Azem, Joel A. Hirsch, Iban Ubarretxena-Belandia

**Affiliations:** 1 Department of Pharmacological Sciences, Icahn School of Medicine at Mount Sinai, New York, NY 10029 USA; 2 Department of Biochemistry and Molecular Biology, School of Neurobiology, Biochemistry and Biophysics, The George S. Wise Faculty of Life Sciences, Tel Aviv University, Tel Aviv, Israel; 3 Instituto Biofisika (UPV/EHU, CSIC), University of the Basque Country, E-48940 Leioa, Spain; 4 Ikerbasque, Basque Foundation for Science, 48013 Bilbao, Spain

**Keywords:** Biochemistry, Structural biology

## Abstract

mHsp60-mHsp10 assists the folding of mitochondrial matrix proteins without the negative ATP binding inter-ring cooperativity of GroEL-GroES. Here we report the crystal structure of an ATP (ADP:BeF_3_-bound) ground-state mimic double-ring mHsp60_14_-(mHsp10_7_)_2_ football complex, and the cryo-EM structures of the ADP-bound successor mHsp60_14_-(mHsp10_7_)_2_ complex, and a single-ring mHsp60_7_-mHsp10_7_ half-football. The structures explain the nucleotide dependence of mHsp60 ring formation, and reveal an inter-ring nucleotide symmetry consistent with the absence of negative cooperativity. In the ground-state a two-fold symmetric H-bond and a salt bridge stitch the double-rings together, whereas only the H-bond remains as the equatorial gap increases in an ADP football poised to split into half-footballs. Refolding assays demonstrate obligate single- and double-ring mHsp60 variants are active, and complementation analysis in bacteria shows the single-ring variant is as efficient as wild-type mHsp60. Our work provides a structural basis for active single- and double-ring complexes coexisting in the mHsp60-mHsp10 chaperonin reaction cycle.

## Abstract (web summary)

The mHsp60-mHsp10 chaperonin system forms alternating single and double ring complexes to assist protein folding, but the molecular details of this cycle are not fully understood. Here, the authors present cryoEM and crystal structures of key intermediates of the mHsp60-mHsp10 reaction cycle.

## Introduction

The group I mitochondrial chaperonin mHsp60 and its co-chaperonin mHsp10 assist the folding of mitochondrial-imported proteins, and correct misfolded polypeptides resulting from mitochondrial stress^1^. Deletion of the mHsp60 gene in yeast is lethal^2^, and inactivation of its homologue in mice leads to embryonic mortality^3^. In addition to their primary protein-folding activity in the matrix, human mHsp60 and mHsp10 have also been implicated in a wide range of extra-mitochondrial and cytosolic activities, including the regulation of inflammatory cytokines, apoptopic processes, and carcinogenesis^4–10^. Several genetic neurodegenerative disorders have also been associated with defects in the human mHsp60–mHsp10 chaperonin system^11–14^.

Human mHsp60 shares ~51% amino acid identity (Supplementary Fig. 1) with the best-studied group I chaperonin, GroEL from *E. coli*^15,16^. Like GroEL, human mHsp60 can also assemble into stacked back-to-back double-heptameric rings capped by a dome-shaped heptameric mHsp10 (GroES in the case of *E. coli*) co-chaperonin lid to form ATP-driven nanocages for a substrate–protein to fold in confinement^17,18^. Despite the high sequence homology, mHsp60 displays notable differences with GroEL in its mechanism of assembly and protein-folding reaction cycle. Whereas GroEL assembles into tight double-heptameric rings even in the absence of nucleotide^19,20^, oligomerization and double-ring formation for mHsp60 depends on ATP^21^. Negative ATP-binding inter-ring cooperativity^22^ characterizes the reaction cycle in GroEL, so that when the *trans*-ring in GroEL binds ATP the *cis*-ring does not. Accordingly, the canonical model for GroEL posits the reaction cycle begins when the GroEL double ring engages the lid formed by GroES, capping one end of the barrel at a time, and where the predominant functional complex is the so-called asymmetric bullet GroEL_14_–GroES_7_^17,18^. In this model, alternation between two forms of bullet complexes, the ADP and the ATP form, characterize the reaction cycle. An alternative model argues that in the presence of substrate–protein the protein-folding cycle also includes the so-called American football GroEL_14_–(GroES_7_)_2_ complexes^23–25^. In this latter model, the reaction cycle comprises alternating double-ring football and bullet complexes.

**Fig. 1:**
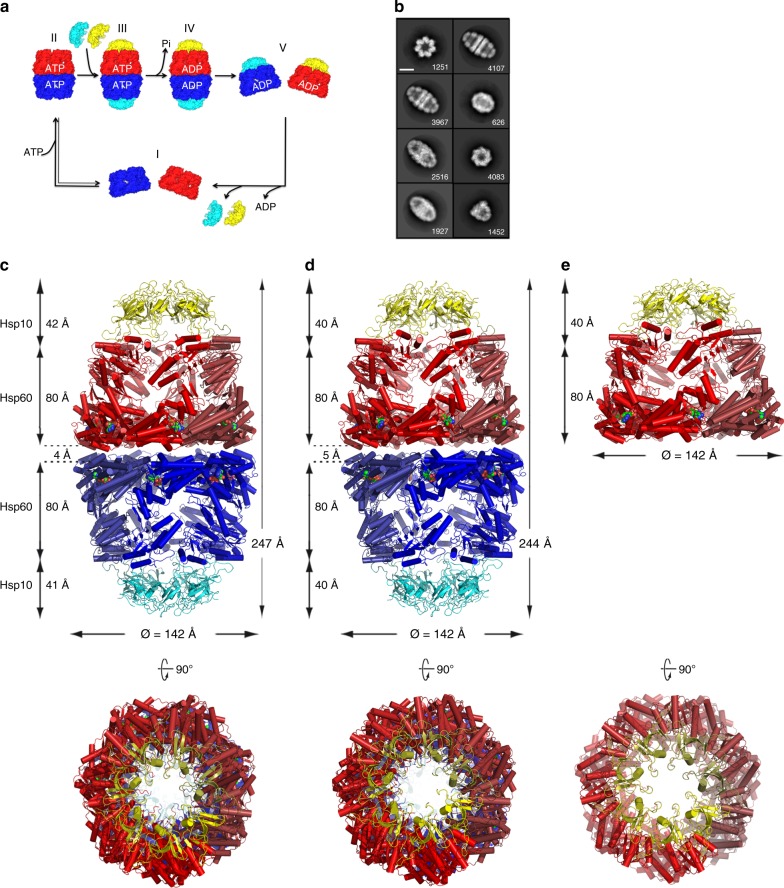
Structures of mHsp60–mHsp10 complexes. **a** Published model for the reaction cycle of mHsp60–mHsp10 (adapted from ref. ^27^). Red and blue denote mHsp60 heptameric north and south rings, respectively. Yellow and cyan denote mHsp10 heptameric north and south lids, respectively. The same color scheme applies to the remaining figure panels. Nucleotide states are labeled. **b** Selected reference-free 2D class averages of mHsp60–mHsp10 complexes show detailed structural features. Side views clearly identify football and half-football complexes. In the classes representing front views, the two complexes can be distinguished by taking into account that the footballs have D7 symmetry (top class left column) and the half-footballs C7 symmetry (third class right column). The numbers show the particles per class, and the scale bar represents 100 Å. **c** Cartoon depiction of the ADP:BeF_3_ bound mHsp60_14_–(mHsp10_7_)_2_ football assembly derived from the 3.7 Å resolution crystal structure viewed down a twofold dyad orthogonal to the sevenfold molecular axis. Below is a top view down the sevenfold axis. Individual mHsp60 subunits are colored in graded hues of the relevant color. Spheres depict the nucleotide. **d** Cartoon depiction of the ADP-bound mHsp60_14_–(mHsp10_7_)_2_ football assembly derived from the 3.08 Å resolution cryo-EM structure. **e** Cartoon depiction of the ADP-bound mHsp60_7_–mHsp10_7_ half-football assembly derived from the 3.83 Å resolution cryo-EM structure. Dimensions of the complexes are, respectively, indicated, using symmetrical Cα atoms to define limiting planes.

In contrast to GroEL, mHsp60 does not display negative ATP-binding inter-ring cooperativity at any point in its reaction cycle^21^. In addition, in the presence of mHsp10 and ATP both single- and double-ring assemblies have been observed^21,26^. Consequently, we postulated a protein-folding reaction cycle devoid of negative cooperativity (Fig. 1a), where both football mHsp60_14_–(mHsp10_7_)_2_ and half-football mHsp60_7_–mHsp10_7_ complexes coexist^21,27^. However, detailed structural evidence for this model was lacking.

We have recently solved the crystal structure (PDB 4PJ1) of a human mHsp60^E321K^_14_–(mHsp10_7_)_2_ variant in the form of a football complex bound to ADP at 3.15 Å resolution^28^. With all mHsp60 subunits in both rings occupied by ADP, we suggested this structure corresponded to a post ATP hydrolysis intermediate representing the complex at an early stage of dissociation. Although this variant cannot progress through the folding cycle, as it is unable to release mHsp10 and liberate the folded substrate–protein^29^, it provided structural evidence for the formation of mHsp60–mHsp10 football complexes.

Here we employ both X-ray crystallography and cryo-electron microscopy (cryo-EM) to solve structures of wild-type (WT) mHsp60–mHsp10 complexes representing a ground-state particle ready for folding, the ADP:BeF_3_-bound double-ring mHsp60_14_–(mHsp10_7_)_2_ football, its apparent successor state, an ADP-bound mHsp60_14_–(mHsp10_7_)_2_ football, and an ADP-bound single-ring mHsp60_7_–mHsp10_7_ half-football, at resolutions ranging from 3.8 to 3.1 Å. Complemented with in vitro and in vivo assays with obligate double and single-ring mHsp60 variants, our work provides a structural basis for active football and half-football complexes coexisting in the reaction cycle of human mHsp60–mHsp10.

## Results

### Structures of mHsp60–mHsp10 reaction cycle intermediates

For cryo-EM we employed functional WT human mHsp60 and mHsp10. Mixing purified mHsp60 and mHsp10 subunits (protomers) at a 1:1.2 molar ratio in the presence of ATP, followed immediately by adsorption onto homemade lacey gold coated grids and vitrification, allowed us to record cryo-EM movies of mHsp60–mHsp10 complexes (Supplementary Fig. 2a) with a detectable signal close to 3.0 Å (Supplementary Fig. 2b). Two-dimensional (2D) classification revealed that mHsp60 and mHsp10 assembled primarily into football complexes (Fig. 1b) accounting for ~70% of the particles, followed by half-football complexes (~30% of the particles) (Supplementary Fig. 2b). These mHsp60–mHsp10 assemblies have been observed before in the presence of ATP^21^. In agreement with previous EM^26^ and biochemical^21^ data, bullet mHsp60–mHsp10 complexes were not observed in any of our images (Supplementary Fig. 2a). Upon image processing and after imposing D7 symmetry, we obtained a three-dimensional (3D) reconstruction (Supplementary Fig. 3; Table 1 displays structure determination statistics) of the mHsp60–mHsp10 football complexes at 3.08 Å resolution (EMD-9195), based on the gold-standard FSC = 0.143 criterion (Supplementary Fig. 4a)^30^. The refined model (Fig. 1d; PDB 6MRC) fits the cryo-EM density map well (Supplementary Fig. 5a, b), where side-chain densities for most amino acid residues of the mHsp60 and mHsp10 subunits were clearly resolved (Supplementary Fig. 5c). Independently of the football complexes, we also determined a 3.83 Å resolution C7 symmetry 3D reconstruction of the half-football complexes observed in this data set (EMD-9196; Supplementary Figs. 4c, 6). The refined model of the half-football is shown in Fig. 1e (PDB 6MRD).

**Table 1:** Cryo-EM data collection, refinement, and validation statistics.

	ADP football (EMDB-9195) (PDB 6MRC)	ADP half-football (EMDB-9196) (PDB 6MRD)
Data collection and processing		
Magnification	22,500	22,500
Voltage (kV)	300	300
Electron exposure (e–/Å^2^)	63	63
Defocus range (μm)	0.8–2.5	0.8–2.5
Pixel size (Å)	1.07	1.07
Symmetry imposed	D7	C7
Initial particle images (no.)	162,669	162,669
Final particle images (no.)	66,013	10,972
Map resolution (Å)	3.08	3.83
FSC threshold	0.143	0.143
Refinement		
Initial model used (PDB code)	4PJ1	6MRC
Model resolution (Å)	3.08	3.83
FSC threshold	0.143	0.143
Map sharpening *B* factor (Å^2^)	140.8	111.6
Model composition		
Non-hydrogen atoms	66,066	33,033
Protein residues	8792	4396
Ligands	14	7
*B* factors (Å^2^)		
Protein	84.6	153.9
Ligand	56.9	125.6
R.m.s. deviations		
Bond lengths (Å)	0.010	0.005
Bond angles (°)	1.25	1.05
Validation		
MolProbity score	2.48	2.69
Clashscore	27.46	44.83
Poor rotamers (%)	0	0
EMRinger score	3.46	0.97
Ramachandran plot		
Favored (%)	89.69	89.77
Allowed (%)	9.84	10.07
Disallowed (%)	0.47	0.16

Biochemical experiments showed that addition of ATP and BeF_3_ generated a stable mHsp60–mHsp10 football complex unable to progress through the reaction cycle and assist substrate–protein folding (Supplementary Fig. 7a). In solution in the absence of ATP size-exclusion chromatography coupled with multi-angle light-scattering (SEC-MALS) showed WT human mHsp60 eluted (Supplementary Fig. 7b, d) mainly as monomers and single heptameric rings (apparent molecular weight of ~445 kDa). In contrast, under the same conditions GroEL eluted (Supplementary Fig. 7b, d) as double-heptameric rings (apparent molecular weight of ~792 kDa). In addition, mHsp10 eluted (Supplementary Fig. 7b, d) as single heptameric rings (apparent molecular weight of ~75 kDa). In the presence of ATP, mHsp10 and mHsp60 eluted as single-ring complexes (Supplementary Fig. 7c, d). However, in the presence of ATP and BeF_3_, which upon ATP hydrolysis yield the ATP ground-state mimic ADP:BeF_3_, mHsp10 and mHsp60 eluted (Supplementary Fig. 7c, d) as oligomers with an apparent molecular weight of ~823 kDa, consistent with a stable double-heptameric ring, likely a football complex. Thus, to obtain the structure of a ground-state mHsp60–mHsp10 football complex, we crystallized WT human mHsp60 and mHsp10 in the presence of ATP and BeF_3_. A diffraction data set with Bragg limits of 3.7 Å enabled us to solve the structure (Fig. 1c; PDB 6HT7) using molecular replacement with a search model based on the 3.08 Å refined cryo-EM structure (Table 2 displays crystallographic statistics).

**Table 2:** Crystallographic data collection and refinement statistics.

Data collection	
Wavelength	0.976 Å
Resolution range	48.95–3.7 (3.832–3.7)
Space group	P 21 21 21
* Cell dimensions*	
*a, b, c* (Å)	141.6, 295.8, 326.6
α, β, γ (°)	90°, 90°, 90°
Total reflections	607,096
Unique reflections	144,762
Completeness (%)	98.7% (98.1%)
Redundancy	4.2
I/σ	6.95 (1.45)
Wilson B factor	120.91
R-merge	14.3%
CC1/2	99.7
Refinement	
Reflections used in refinement	144523 (14233)
Reflections used for R-free	1998 (197)
R-work	0.2421 (0.3472)
R-free	0.2905 (0.3665)
Number of atoms	
Macromolecules	65,568
Ligands	462
Protein residues	8791
RMSD	
Bonds (Å)	0.009
Angles (degrees)	0.98
Average B factor (Å^2^)	
Macromolecules	146.67
Ligands	111.59

Statistics for the highest-resolution shell are shown in parentheses.

The near-atomic resolution structures of these three assemblies are depicted in Fig. 1c–e in the order of the postulated reaction cycle (Fig. 1a), i.e., stages III through V. In the two football complexes, their respective football halves we term the north pole and south pole. Features shared by all three structures include mHsp60 protomers in an extended conformation along the molecular symmetry axis with clearly defined domains, namely an equatorial ATP-binding domain (residues 1–137, 411–526), an intermediate hinge domain (residues 138–191, 375–411), and an apical domain (residues 192–374) (Supplementary Fig. 5b). All mHsp60 rings are bound to mHsp10 lids, where mHsp10 appears in the obligate heptameric form^31^ (Supplementary Fig. 7b, d), with little conformational variation between subunits (RMSD < 0.3 Å; Supplementary Table 1). Each mHsp10 protomer adopts the canonical seven-strand β-barrel structure and exposes a flexible loop sequence of twenty residues (mobile loop) that mediates the interaction with helices H and I of the mHsp60 apical domains. This mHsp10 lid conformation is rigid with low RMSD superposition values between all structures (RMSD < 0.5 Å; Supplementary Table 2).

**Fig. 2:**
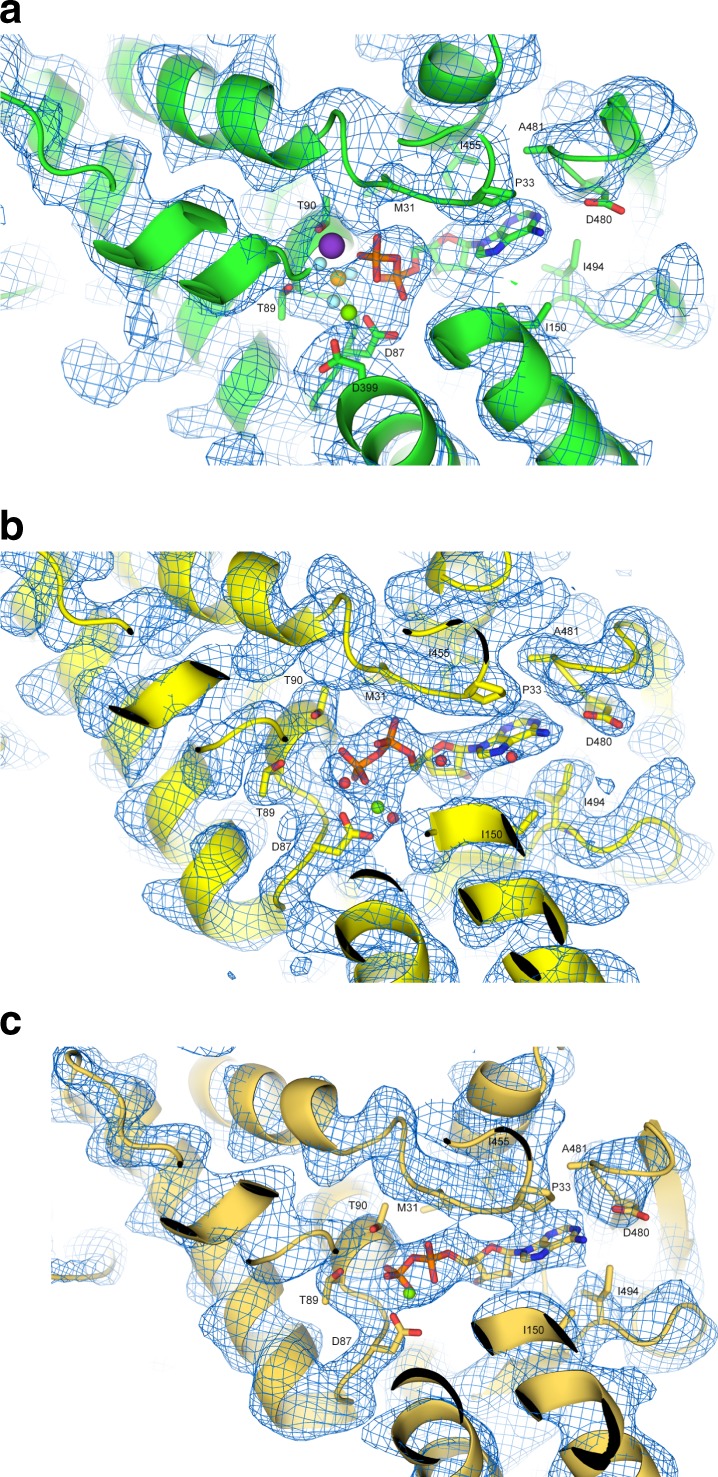
Nucleotide state of the complexes. **a** ADP:BeF_3_-bound football complex. Electron-density omit map at 1σ and the atomic model are shown around the nucleotide-binding site with one bound ADP molecule and BeF_3_. Selected residues in the nucleotide binding pocket are indicated. The Mg^2+^ ion is marked as a green sphere, K^+^ as purple, and BeF_3_ as gold surrounded by cyan spheres. **b** ADP-bound football complex. Details of the cryo-EM density and the atomic model around the nucleotide-binding site with one bound ADP molecule are shown. Selected residues in the nucleotide binding pocket are indicated. The Mg^2+^ ion is marked as a green sphere, and water molecules are represented as red spheres. **c** ADP-bound half-football complex. Details of the cryo-EM density and the atomic model around the nucleotide-binding site with one bound ADP molecule. Selected residues in the nucleotide-binding pocket are indicated. The Mg^2+^ ion is marked as a green sphere.

Density features in the nucleotide-binding sites allowed us to unambiguously identify the nucleotide state of all mHsp60–mHsp10 complexes (Fig. 2). For the ground-state football crystal structure (stage III, Fig. 1c), we modeled ADP:BeF_3_, Mg^2+^ and K^+^ only at later stages of refinement where the density was most consistent with such an interpretation. *Fo–Fc* difference maps display strong positive peaks when ADP alone is modeled. At the current resolution, it is not possible to precisely place atoms based on the electron density features but the density for the pocket, also seen with omit maps (Fig. 2a), support our modeling of the BeF_3_, Mg^2+^, and K^+^ moieties. All nucleotide pockets in this complex appear alike, and we modeled them all as occupied by ADP:BeF_3_. For the ADP football (stage IV, Fig. 1d), we conclude with confidence, as a result of the high resolution and quality of our cryo-EM map, that the nucleotide-binding sites in all fourteen mHsp60 subunits in both symmetric halves of the football are occupied by ADP and Mg^2+^, with no evidence for the presence of γ phosphate or K^+^ (Fig. 2b). The specific amino acid interactions with ADP and Mg^2+^ mirror those reported in the ADP-bound crystal mHsp60^E321K^–mHsp10 structure^28^, except that in our map several water molecules are also visible. The same ADP-bound nucleotide state (Fig. 2c) is found in the half-football (stage V, Fig. 1e).

### Intra-ring subunit interface

mHsp60 exhibits an ATP-dependent monomer/heptamer/tetradecamer oligomeric equilibrium in a protein concentration-dependent manner^21^. What is the structural basis for this equilibrium? One region, structurally conserved in all three current structures, is the equatorial domain β-sheet formed from the N-terminal and C-terminal β-strands of one subunit, and a β-hairpin from the neighboring subunit (Supplementary Fig. 8a). This area, stabilized by both main chain and side-chain H-bonds and VDW interactions, is proximal to the nucleotide-binding pocket and the position of the γ phosphate, suggesting a structural means by which ATP binding might assist ring formation^21^. Consistent with this structural observation, a three-residue N-terminal truncation abrogates the need for nucleotide in ring formation while a pathogenic point mutant D3G destabilizes it^32^. An additional interface point is found in the apical domains; namely residues of helix K from one subunit interact with a loop between helix G2 and strand seven of its neighbor. Helices K and L form a helical hairpin, a so-called K/L protrusion^18,33^ that makes up a substantial portion of the wall of the folding cavity. The positioning of the interaction varies from interface to interface along a face of helix K in the ground-state complex, a source of subunit conformational asymmetry, while it is fixed in the ADP football (Supplementary Fig. 8b, c) in a manner that minimizes the window of the mHsp60 folding cavity.

Finally, the C-terminal 22 amino acid-long tail ending with two GGM repeats has been proposed in GroEL to protrude from the equatorial domains into each ring cavity, thereby occluding free passage between cavities^34^. While density for the complete GGM tail is not visible in our structures, in line with the fact that this region is predicted to be unstructured, we did observe extended density radiating into the center of the ring for the ADP football. We modeled several residues not visible for the ground state structure, suggesting a graded ordering of this barrier between cavities as the reaction cycle progresses.

### Transition from subunit conformational asymmetry to symmetry

**Fig. 3:**
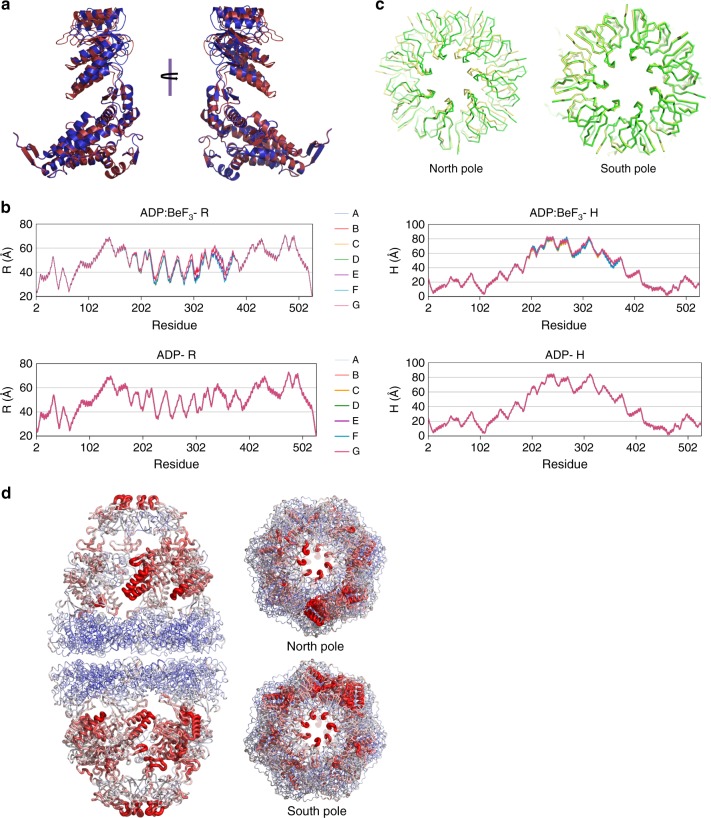
Transition from the ATP ground-state mimic to ADP football. **a** Superposition of the two most conformationally divergent mHsp60 subunits from the ADP:BeF_3_-bound football complex shows significant backbone conformational asymmetry. The two perspectives depicted are related by 180° rotation. **b** The crystal structure of the ADP:BeF_3_-bound football complex and the structure of the ADP-bound football complex built from the C1 cryo-EM map were subjected to a conformational analysis using a cylindrical coordinate system previously employed to analyze the conformation of individual subunits in GroEL^36,37^. In this analysis, R is the distance between the Cα of a given residue and the sevenfold symmetry axis, whereas H is defined as the height of the Cα of a given residue over the twofold plane of symmetry between two rings. Each trace represents one mHsp60 subunit within the same ring. From top to bottom: ADP:BeF_3_–R: radial distance to the longitudinal axis for the ADP:BeF_3_-bound football complex crystal structure; ADP–R: radial distance for the ADP-bound football complex C1 cryo-EM structure; ADP:BeF_3_–H: height from the equator of the complex for the ADP:BeF_3_-bound football complex crystal structure; ADP–H: height from the equator for the ADP-bound football complex C1 cryo-EM structure. **c** Top views of superposed ADP:BeF_3_ and ADP footballs in green and yellow, respectively. The north equatorial domains of mHsp60 were the templates for superposition. The rotation of the mHsp10 lid is clearly seen in the north pole, while in the south pole the structures coincide with little deviation. **d** B-factor analysis of the ground-state ADP:BeF3 crystal structure football complex. B factors are depicted by color and thickness of the worm representation. Blue and thin indicates low values while red and thick indicates high values, respectively. Side, south, and north pole views are shown.

Structural studies of GroEL have shown that its subunits undergo large conformational changes driven by ATP binding and hydrolysis along the reaction cycle (reviewed in ref. ^20^). These conformational changes have been proposed to progress via structural intermediates arising from the concerted movement of all seven apical domains within one ring^22,35^. This concerted movement leads to conformational symmetry among identical subunits. Notably, the ADP:BeF_3_ football structure presented here displays subunit conformational asymmetry, as determined from a superposition analysis of all the mHsp60 subunits (Supplementary Table 3) and consistent with our inability to perform non-crystallographic averaging (see “Methods”). Subunit conformational variability in the ADP:BeF_3_ football ranges from 0.3 to 2.2 Å RMSD (Fig. 3a), and largely localizes to the intermediate and apical domains. In addition, we performed an alternate conformational analysis of the football structures using a cylindrical coordinate system previously employed to analyze the conformation of individual subunits in GroEL^36,37^. In this analysis, R is the distance between the Cα of a given residue and the sevenfold symmetry axis, while H is defined as the height of the Cα of a given residue over the twofold plane of symmetry between two rings. As seen in Fig. 3b, we can discern significant asymmetry in the intermediate and apical domains for the ADP:BeF_3_ football structure. The observed subunit conformational asymmetry does not appear to be caused solely by crystal packing, since subunits not involved in crystal contacts nonetheless vary from each other.

We examined this same question for the ADP football structure determined by cryo-EM. To this end, we built a refined structure model from a 3D reconstruction at 3.74 Å resolution determined without imposing any symmetry (C1 symmetry) (Supplementary Fig. 9). The conformational analysis described above (Fig. 3b) showed the unsymmetrized cryo-EM structure displays perfect symmetry, indicating that in solution the ADP-bound football does not display subunit conformational asymmetry. Together, our analysis shows a transition from the ADP:BeF_3_-bound football complex, displaying significant subunit conformational asymmetry, to a conformationally symmetric ADP-bound football complex. This transition along the reaction cycle might be a unique feature of the human mitochondrial chaperonin.

A dramatic structural change in the transition from the ADP:BeF_3_-bound ground-state mimic to the ADP-bound football occurs with a rotation of the north pole mHsp10 lid between seven and fifteen degrees (Fig. 3c). This rotation or twisting is not uniform since the ground-state football itself is conformationally asymmetric, and is observed when using the north equatorial domain as the template for superposition. Strikingly, the south pole mHsp10 lid aligns almost perfectly (Fig. 3c). This twist is enabled by the plasticity of the apical domains in their interface with the mHsp10 mobile loops.

Next, we sought insight into the dynamic properties of the assemblies, since such information is likely to point to mobile or flexible structural components in the cycle. When we mapped the individual isotropic B-factors on the ground-state football (Fig. 3d), we discovered that the equatorial domain in mHsp60 is most rigid, while the apical domain is most flexible, along with mHsp10. For the ADP:BeF_3_ football, there is a rotationally graded B-factor asymmetry in apical domains around the sevenfold axis, with different polarity for each pole. In addition, the mHsp10 β-barrel mobility is diminished compared to mHsp60 apical domain sections. We conclude that the subunit conformational asymmetry observed for the ground state football may represent snapshots of different dynamic subunit conformations, which are consistent with the B-factor analysis and provide a basis for an intra-ring communication mechanism akin to that observed for CCT^38^.

### Unique inter-ring interface rearranges in the reaction cycle

The structures of the ADP footballs and half-footballs provide potential evidence for a second level of structural dynamics along the reaction cycle, namely the inter-ring split of mHsp60–mHsp10 football complexes. To understand the structural basis for this possible pathway, we performed a comparison of several structural parameters between the structures of the three reaction cycle intermediates. The differences between the ADP football and half-football are quite small (RMSD ~0.5 Å), and indicate that these structures are essentially the same, saving for the quaternary structure difference (football vs. half-football).

In contrast, when comparing the ADP:BeF_3_ football to ADP football, the RMSD values range from 0.7 to 1.8 Å, respectively (Supplementary Table 4), mainly due to significant conformational changes in the apical and intermediate domains of the mHsp60 subunits. The volume of the ground-state football is 2.5% larger than the ADP football, where it is also 3 Å longer along its sevenfold axis. The buried surface area for all the individual halves (mHsp60_7_–mHsp10_7_) are about the same ~6400 Å^2^, except for the south half of the ground-state football, again emphasizing this assembly’s subunit asymmetry (Supplementary Table 5). Notably, the inter-ring interface buried surface area for the ADP:BeF_3_ ground state mimic is markedly larger (>1000 Å^2^) than for the ADP football. This differential points to perhaps the most critical divergence of the two football structures, namely the geometry and interactions of the inter-ring interface.

**Fig. 4:**
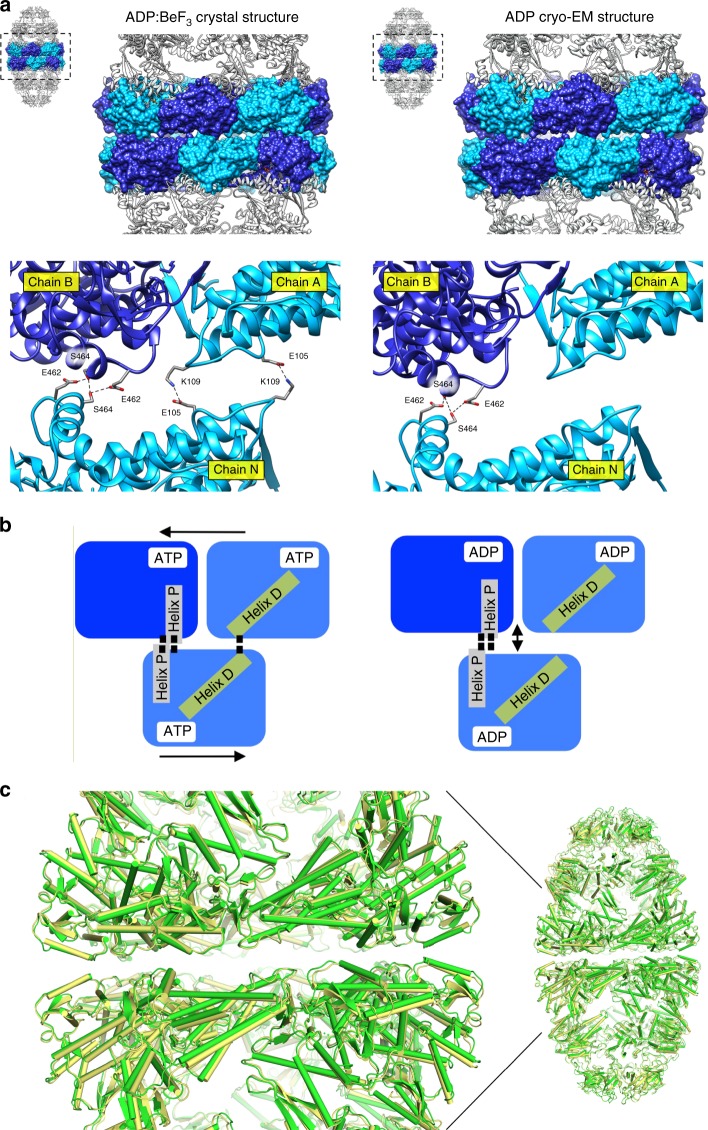
mHsp60 inter-ring interface of mHsp60–mHsp10 football complexes. **a** Side views of the ADP:BeF_3_ (left) and the ADP (right) footballs. The equatorial domains of the mHsp60 subunits are rendered as a molecular surface in alternating blue and light blue. The other domains in the mHsp60 subunits and mHsp10 subunits are presented as ribbon diagrams. Below is a close-up view of the staggered arrangement of two mHsp60 subunits in one ring contacting one subunit in the opposite ring represented as ribbon diagrams. Residues engaged in inter-ring contacts are presented as sticks. In the ADP:BeF_3_ football, these are E105, K109, E462, and S464 (left). In the ADP football, these are S464 and E462 (right). Black dashed lines denote bonds between the interacting residues. **b** Cartoon representing the differences in the interactions between left (Helix D) and right (Helix P) contact sites of the mHsp60 subunits across each ring of the ADP:BeF_3_ (left) and ADP (right) footballs. **c** Superposition of the ADP:BeF_3_ (green) and ADP (yellow) football structures showing a close-up of the mHsp60 inter-ring region.

In the ground-state football (Fig. 4a), two loci of interactions stitch the rings together, dubbed right and left (perspective from inside the assembly). The right locus involves a twofold symmetric interaction between S464 residues of apposite rings, buttressed by a H-bond from E462. This H-bond interaction has not been observed in GroEL, which relies at the right locus on an inter-ring salt bridge between the residues E461 and R452^18^. The left locus involves a salt bridge from residues located on the loop between helices D and E, K109 and E105 of interfacing subunits. The corresponding inter-ring residues in GroEL are K105 and A109 that interact by virtue of the helix dipole of helix D^39^. We note that helix D’s N-terminus forms the nucleotide-binding pocket (schematically depicted in Fig. 4b) and the loop preceding it binds the nucleotide γ phosphate (or its equivalent here, BeF_3_). Moreover, in the ADP:BeF_3_ football the equatorial gap between the two rings is 3.5 Å, as measured from planes delimited by the Cα of mHsp60 residue 465. In contrast, the ADP football has lost the left interaction locus, and the double mHsp60 rings are held together solely by the S464 interaction. Concomitantly, the equatorial gap has increased to 4.6 Å with the buried surface area diminished, consistent with a football poised to split into its two halves. Further, when we superimposed the ADP:BeF_3_ and ADP football structures (Fig. 4c), we discern a subtle but distinct concerted pivot of the ADP equatorial domains away from the interface plane, abrogating the left locus and giving rise to the single point S464 interaction. Biochemical and structural evidence has shown that communication between two GroEL rings is crucial for GroES release^40^, and that the ionic interactions at both left and right contact sites are involved in the transmission of the allosteric signal^41,42^. Our structural evidence for mHsp60–mHsp10, however, points to minimal contacts holding the two mHsp60 rings together (Fig. 4b), in a manner consistent with the lack of inter-ring negative cooperativity observed in biochemical experiments^21^.

**Fig. 5:**
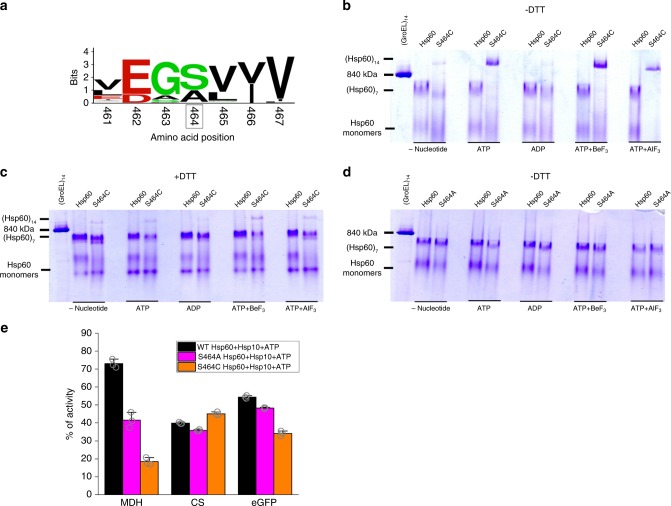
Residue S464 plays a pivotal role in the assembly of mHsp60 double rings. **a** Logo depiction of amino acid frequency of the respective residue positions in 120 mHsp60 orthologue sequences. Amino acids are colored according to their chemical properties: polar amino acids are green, basic blue, acidic red, and hydrophobic amino acids are black. **b**, **c** Native gel analysis of WT mHsp60 and the S464C variant assembly behavior, under oxidizing (labeled − DTT) and reducing (labeled + DTT) conditions. In the experiments with nucleotide, it was added only during the pre-incubation with the chaperonin, but omitted from the loading buffer, gel, and running buffer, and thus nucleotide is effectively depleted during the electrophoresis due to separation from the protein pool and hydrolysis. **d** Native gel analysis of WT mHsp60 and S464A variant protein assembly behavior, under oxidizing (−DTT) conditions. The gels are representative of at least two independent experiments. **e** Refolding of substrate proteins MDH, CS, and D116/G117-eGFP by WT mHsp60 and its listed variants. Data represent average ± s.d. from three independent experiments (*n* = 3). Source data are provided as a Source Data file.

Given these structural findings, we tested whether the S464 residue is a critical interactor mediating the assembly of mHsp60 double rings. A bioinformatics analysis of 120 mHsp60 orthologue sequences showed the amino acid serine to be conserved at this position, followed by alanine (Fig. 5a). Previous studies demonstrated that when ATP was readily present at all steps during electrophoresis^28^, or in the presence of a chemical cross-linker^32^, the shift to double rings in mHsp60 can be detected by native gels. Under conditions where ATP or its analogues are present only during pre-incubation, but omitted from all subsequent electrophoresis steps, the S464C mHsp60 mutation prevented inter-ring separation (Fig. 5b). This stabilization was due to disulfide formation during the pre-incubation, as inferred from the absence of double-ring stabilization in the presence of DTT or for the S464A variant (Fig. 5c, d). Next, we examined the ability of S464A and S464C variants to assist the refolding of model substrates (Fig. 5e). Both variants were able to assist the folding of citrate synthase (CS) to a level comparable to that displayed by WT mHsp60, but were impaired in their ability to assist the folding of malate dehydrogenase (MDH). Notably, the S464C variant was also impaired in its ability to assist the folding of a circular permutant (D116/G117) of enhanced green fluorescent protein (D116/G117-eGFP), shown to strictly depend on GroEL for its in vitro refolding^43^. These data suggest that substitution of amino acid residue S464 may affect negatively the ability of mHsp60 to assist the refolding of certain substrates.

### Functional single and double mHsp60 ring complexes

**Fig. 6:**
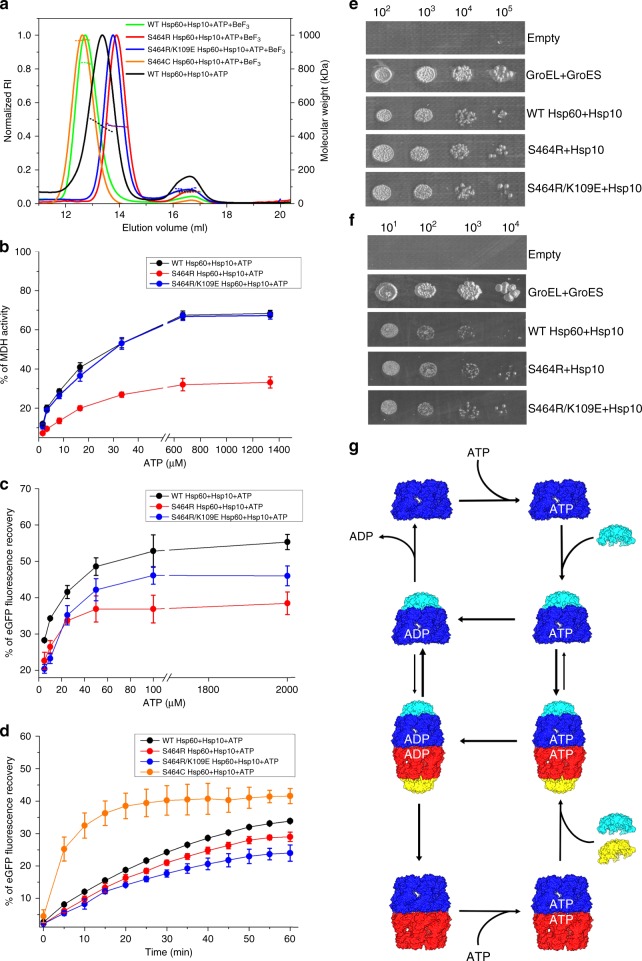
Obligate single mHsp60 rings assist folding in vitro and in vivo. **a** SEC-MALS profiles of WT mHsp60–mHsp10 (green trace, average MWs of first and second peaks are 823 and 68 kDa, respectively), S464C mHsp60–mHsp10 (orange trace, average MW of first peak is 970 kDa), S464R mHsp60–mHsp10 (red trace, average MWs of first and second peaks are 464 and 67 kDa, respectively), and S464R/K109E mHsp60–mHsp10 (blue trace, average MWs of first and second peaks are 464 kDa and 77 kDa, respectively). The proteins were pre-incubated with 1 mM ATP, 1 mM BeCl_2_, and 10 mM NaF for 5 min. The profile of WT mHsp60–mHsp10 pre-incubated with 1 mM ATP for 5 min is shown in black trace (average MWs of first and second peaks are 462 and 73 kDa, respectively). The SEC profiles are plotted against the normalized refractive index (RI) and the expected molecular mass (dashed lines). **b**, **c** Refolding of MDH and D116/G117-eGFP substrates by WT and single-ring mHsp60 variants in the presence of ATP and mHsp10. All the refolding experiments were performed at 37 °C, and data represent average ± s.d. **d** Refolding of D116/G117-eGFP under oxidizing conditions by WT mHsp60 and its listed variants. Data represent average ± s.d. from three independent experiments (*n* = 3). **e**, **f** Tenfold serial dilutions of GroEL–GroES-deficient *E. coli* strain MGM100 transformed with plasmid pOFX bearing the indicated mHsp60 mutants and WT mHsp10. Colonies were grown on agar plates in the presence of glucose and IPTG at 30 °C (**d**) and 37 °C (**e**). **g** Revised model for the reaction cycle of mHsp60–mHsp10 based on the findings of this study. Color coding is the same as in Fig. 1a. Source data for panels a-d are provided as a Source Data file.

As mentioned above, while active GroEL–GroES is invariably found in the double-heptameric ring form^19,20^, mHsp60 can be found as single- and double-ring species^21^. Our SEC-MALS analysis (Supplementary Fig. 7) and the cryo-EM structures of the ADP football and half-football (Fig. 1b, c) complexes are consistent with these findings. Here we addressed the question of whether single- and double-ring complexes could function independently from each other, or if alternation between them was necessary for mHsp60–mHsp10 folding activity. To this end, we generated inter-ring S464R and S464R/K109E mHsp60 variants, which based on our football structures we predicted would prevent double ring formation. SEC-MALS analysis confirmed that in the presence of ATP and BeF_3_, under conditions where WT mHsp60 and the S464C variant formed stable football complexes with mHsp10, both S464R and S464R/K109E mHsp60 variants assembled into single rings without evidence of higher oligomeric species (Fig. 6a). Next, we examined the ability of S464R and S464R/K109E mHsp60 single-ring variants to assist the refolding of MDH and D116/G117-eGFP as a function of ATP concentration (Fig. 6b, c). The S464R single-ring variant was significantly impaired in its ability to refold MDH and D116/G117-eGFP, while the S464R/K109E single-ring variant was impaired in its ability to refold only D116/G117-eGFP. Thus, although reduced compared with WT mHsp60, both these obligate single-ring variants displayed significant activity against these substrates. Notably, the mHsp60 S464C cysteine-trap variant, shown above to be an obligate double-ring complex in the absence of DTT, displayed a significantly higher rate of refolding of D116/G117-eGFP compared with WT mHsp60 and the obligate single-ring S464R and S644R/K109E variants (Fig. 6d). Taken together these data confirm that half-football and football complexes can assist in vitro folding independently of each other, while the double ring complexes can be more active.

We next examined whether the single-ring mutants are able to complement the GroEL–GroES deleted *E. coli* MGM100 strain, with the caveat that the native environment of mHsp60 is the human mitochondria and not bacteria. Both these single-ring mutants were as competent as WT mHsp60 in sustaining cell growth at 30 and 37 °C (Fig. 6e, f), indicating that half-footballs can also function in vivo independently of footballs. Overall, we conclude that double-ring complexes might provide advantage in vitro under limiting conditions for certain substrates, however, there appears to be no advantage for WT mHsp60 compared with the single-ring mutants in *E. coli*.

## Discussion

This work represents a major advance toward unraveling the structural basis underlying the function of group I chaperonins, as it reveals views of a WT double-ring mHsp60–mHsp10 football complex mimicking the ATP-bound ground-state intermediate and the changes in symmetry and at the inter-ring interface resulting from ATP hydrolysis. We also provided structural details of half-football complexes, and experimental evidence suggesting that active single- and double-ring mHsp60 complexes coexist in the reaction cycle of the human mHsp60–mHsp10 chaperonin system.

Specifically, our work provides a structural basis for three unique aspects of the reaction cycle of mHsp60–mHsp10. First, it was known that nucleotides regulate the oligomeric state of mHsp60 tetradecamers^44^. In contrast to GroEL, ATP in mHsp60 serves as a molecular linchpin for heptamer assembly and subsequent association of heptameric rings. Based on the structural changes observed along the reaction cycle and the effect of nucleotide on the oligomeric state of the variant S464C, we propose that in the context of the steady-state (i.e., 1 mM ATP concentration as found in the cell) ATP modulates oligomerization constants thus shifting the equilibrium toward the double-ring state. Indeed, the nucleotide’s primary function may be inducement of oligomerization, as opposed to providing energy for apical and intermediate domains’ conformational shift, as observed in GroEL^45^.

Second, the labile nature of mHsp60 oligomers, in particular between rings, points to a system that employs a mechanism devoid of ATP-binding negative inter-ring cooperativity. Indication for such a possibility was initially obtained from analyzing the ATPase activity of the system^21,26,29^. In the case of GroEL, negative cooperativity is supported structurally by the fact that under native conditions, in all structures reported, the same nucleotide type never occupies the two rings. The two exceptions are crystal structures of ground-state football GroEL obtained in the presence of BeF_3_^37^ or using a variant defective in ATP hydrolysis^46^. For cryo-EM, we mixed mHsp60 and mHsp10 in the presence of ATP, followed immediately by adsorption onto a grid and vitrification. However, all the nucleotide-binding sites in our football and half-football WT mHsp60–mHsp10 structures were occupied by ADP, indicating that under our specimen preparation conditions ATP hydrolysis had occurred rapidly. The homogeneous distribution of nucleotides among subunits provides direct evidence for the absence of negative cooperativity between rings of mHsp60, and indicates the ability of the rings to act as independent engines. Despite the homogenous nucleotide identity, the conformation of the subunits within and between rings of the ground state is different and exhibits conformational asymmetry. Subsequently, in the transition from the ground state (ATP football) to the ADP football, all subunits shift to both nucleotide and conformationally identical states. We argue that this transition to subunit symmetry might be related to the accepted notion in the field of protein oligomerization, that a symmetric assembly is at a lower energy state than its asymmetric counterpart^47,48^. In the context of mHsp60, ATP hydrolysis will be associated with asymmetry, but subsequently the system relaxes into the symmetric and lower energy ADP-bound state.

Third, previous in vitro analysis suggested that mHsp60 undergoes inter-ring split (also termed equatorial split) during its reaction cycle^21,26^. However, at which stage in the reaction cycle of mHsp60 would the rings separate was unknown. Recently, a possible equatorial split has also been reported for GroEL^49^, although the necessity of ring splitting for the function of GroEL is still controversial, as linking of both rings by either formation of S–S bonds^49^ or covalent fusion still allows for significant protein-folding activity^50^. From our cryo-EM images showing abundant ADP-bound football and half-football complexes, we postulated the former might represent a pre-split intermediate. Native gel analysis of the cysteine-trap variant (S464C) provided direct evidence for the modulation of single ring to double ring structures by nucleotides. Clearly, in the absence of nucleotides or the presence of ADP, the mHsp60 molecules assemble into single-ring structures, while in the presence of ATP or its non-hydrolysable analogues it forms double-ring structures.

In order to test whether the validity of our hypothesis that the functional cycle proceeds via an equatorial split comprised of alternating single-ring and double-ring assemblies, we generated two types of variants. One type consists of obligate single-ring mHsp60 (S464R and S464R/K109E variants), while the other consists of obligate double rings (S464C cysteine-trap variant). Experiments with the single-ring variants demonstrate that while under certain conditions they are less efficient in assisting substrate–protein folding, they are nonetheless functional without the requirement to form double rings.

Obligate mHsp60 double-ring assemblies were trapped by formation of inter-ring disulfide bonds in the absence of reducing agent (Fig. 5b). If inter-ring split is essential then the refolding activity of the complex ought to be compromised in the absence of reducing agent, but not in its presence. Strikingly, the cysteine-trap variant was even more active than WT and the two single-ring variants in the absence of reducing agent. Hence, our results indicate that both single and double rings are functional and that the equatorial split is not essential for the folding activity of the mHsp60–mHsp10 chaperonin system, in vitro, or in bacteria.

In light of these findings, we have updated our initial model to include two separate folding cycles with single-ring and double-ring pathways, with the potential of crossing over upon equatorial split of the double rings (Fig. 6g). In this model, the occupancy of nucleotide-binding sites by ADP triggers the dissociation of mHsp10, consistent with reports showing that mHsp60 binds mHsp10 in the presence of ATP, AMPNP and ADP-BeF_3_, but not ADP and ADP-AlF_3_^21,26^. In line with mHsp60 nucleotide-dependence studies^21,29^, exchange of ADP would occur in the presence of excess ATP.

These separate cycles then raise the question as to what might be the biological advantage of two possible modalities for mHsp60–mHsp10, i.e., assisted folding by way of footballs or half-footballs. We speculate that the individual cycles may be optimized for different substrate sets, thereby expanding and tuning this chaperonin system for its host of substrates in mitochondria, in an analogous manner as recently suggested for *E. coli* GroEL–GroES^51^.

## Methods

### Protein expression and purification

mHsp60 is translated in the cytosol as a 573 amino acid-long polypeptide containing a 26 amino acid-long mitochondrial Matrix Targeting Signal (MTS), which when cleaved yields the mature 547 amino acid-long mitochondrial protein (Supplementary Fig. 1a). Human mt-cpn60 gene, lacking the N-terminal mitochondrial MTS, was cloned into a modified pET21d plasmid^52^. The N-terminal HisTagged protein with an engineered TEV cleavage site was overexpressed in *E. coli* Rosetta^TM^ strain for 3 h at 25 °C following induction by 1 mM IPTG at OD_600_ = 0.6. The harvested cells were suspended (1:10 w/w) in buffer (100 mM Tris-HCl pH 7.7, 10 mM MgSO_4_, 1 mM β-ME, 5% glycerol, 0.1% Triton X-100 and protease inhibitors) and lysed using a microfluidizer. Following centrifugation for 30 min at 35,000x*g*, the supernatant was diluted (1:3 w/w) in buffer (10 mM Tris-HCl pH 7.7, 5% glycerol, 0.1% Triton X-100, and 10 mM imidazole), loaded onto a nickel–agarose resin column (equilibrated with the latter buffer) and eluted with 250 mM imidazole. Fractions containing mt-cpn60 were pooled and, upon addition of TEV protease (1:30 w/w), dialyzed overnight against 20 mM Tris-HCl pH 7.7, 5% glycerol, and 200 mM NaCl at 4 °C. As a result of TEV cleavage and removal of the HisTag, the mHsp60 employed for structural studies begins with the amino acid sequence GS. Next, the protein sample was diluted with sixfold volume of 20 mM Tris-HCl pH 7.7 and 12-fold volume of H_2_O, loaded onto an anion-exchange column (equilibrated with 20 mM Tris-HCl pH 7.7, 5% glycerol, 200 mM NaCl, and 10 mM imidazole), and eluted with a 100–400 mM NaCl gradient. Fractions containing monomeric mHsp60 were collected and concentrated. The concentrated sample (18 mg/ml) was then incubated at 30 °C in the presence of 4 mM ATP, 20 mM KCl, and 20 mM Mg acetate, in order to induce oligomerization of monomers. After 2 h, the sample was loaded on a Superdex 200 gel-filtration column in 50 mM Tris-HCl pH 7.7, 300 mM NaCl, and 10 mM MgCl_2_. Fractions containing active oligomeric mHsp60 were collected, concentrated, and frozen using liquid nitrogen. GroEL and GroES were overexpressed in *E. coli* and purified by a combination of size-exclusion and ionic exchange chromatography^53^.

Human mt-cpn10 was expressed without a HisTag (Supplementary Fig. 1b)^28^. Bacterial cell pellet was resuspended (1:10 w/v) in a buffer containing 20 mM Tris-HCl pH 7.7, 5 mM MgSO_4_, 1 mM DTT, PMSF (0.5 mM), and 1 μg/ml each of the protease inhibitors pepstatin, chymostatin, antipain, leupeptin, and aprotinin (Sigma), and 1500 units of DNase and lysed by sonication. Cellular debris was removed by centrifugation for 30 min at 35,000x*g*. The supernatant was loaded onto a RESOURCE Q column (GE Healthcare) equilibrated with buffer A (20 mM Tris-HCl pH 7.7, 0.1 mM EDTA, and 1 mM DTT). Eluted proteins were collected and dialyzed overnight against buffer B (20 mM MES pH 6.6 and 0.1 mM EDTA). The protein was then loaded on a SOURCE-S column (GE Healthcare) equilibrated with buffer B. Bound proteins were eluted from the column with a linear gradient of 0-500 mM NaCl. mHsp10-enriched fractions were collected and concentrated. The protein was then loaded on a Superdex 200 prep grade gel-filtration column (Pharmacia) equilibrated with buffer C (50 mM Tris-HCl pH 7.7 and 100 mM NaCl). Afterward, purified mHsp10 was concentrated and flash-frozen in liquid nitrogen for storage.

For purification of D116/G117-eGFP bacterial pellets (Rosetta™ strain (Merck)) were suspended in 50 mM Tris buffer (pH 7.5) containing 100 mM NaCl, 10 mM imidazole, and 0.1 mM DTT^54^. The cells were then disrupted using sonication, and the lysate was clarified by centrifugation. The supernatant was loaded onto a 5 ml Nickel column, and D116/G117-eGFP was eluted with 200 mM imidazole. The eluted protein dialyzed, and afterward loaded onto Q column and eluted with gradient of 0.01–1 M NaCl. Concentrated D116/G117-eGFP was loaded onto a Superdex 75 column (Amersham Pharmacia) equilibrated with 10 mM phosphate buffer (pH 7) containing 0.1 mM DTT. Fractions containing D116/G117-eGFP were combined, divided into aliquots that were snap-frozen in liquid nitrogen, and stored at −80 °C.

### Crystallization

The football complex was made by mixing 215 μM mHsp60, 455 μM mHsp10, 1 mM ATP, 1 mM BeCl_2_ and 10 mM NaF in Buffer F (75 mM Tris pH 7.7, 15 mM MgCl_2_, 150 mM KCl, and 300 mM NaCl). Crystals containing mHsp60 and mHsp10 were initially grown in 10% PEG 6000 and 2 M NaCl at 293 K. Optimized, diffraction-quality, crystals were grown in 10% PEG 6000, 2.1 M KCl, and Na-HEPES buffer pH 8. Crystals prepared for diffraction measurements were mounted in cryo-loops, frozen in the cryostream, removed, and then soaked in cryoprotectant buffer (10% PEG 6000, 2.1 M KCl, Na-HEPES buffer pH 8 and 20% glycerol) for 5 min and then plunged into liquid nitrogen.

### Data collection and crystallographic structure determination

Diffraction data of the mHsp60–mHsp10 ADP-BeF_3_ complex crystals were collected at beamline ID29 at the European Synchrotron Radiation Facility (Grenoble, France). 1140 images with 0.1° oscillation were collected at 100 K. The data were indexed, integrated, and scaled using XDS^55^. The structure was solved by molecular replacement using PHASER implemented in the Phenix software suite^56^. The search model was a half-football mHsp60–mHsp10 ADP complex derived from the football mHsp60–mHsp10 ADP complex determined by single-particle cryo-EM, as described below, with the nucleotide removed. Subsequently, molecular replacement searches employed a heterodimeric mHsp60–mHsp10 structure, again derived from the cryo-EM determined mHsp60–mHsp10 ADP football complex. Both searches concluded with a football structure solution. Refinement proceeded with this model. In the first stage, rigid-body refinement was performed, dividing the mHsp60 protomer into three domains, equatorial, intermediate, and apical along with mHsp10. This round improved the electron-density maps significantly. Notably, NCS averaging degraded the maps considerably. Hence, strict NCS constraints were not used for ensuing refinement but rather NCS restraints. Rounds of standard refinement (phenix.refine) including TLS methods and manual rebuilding using Coot^57^ produced a model of good stereochemistry and R-factors.

### Electron microscopy

Aliquots of purified mHsp60 and mHsp10 were diluted to 50 μM in reaction buffer (20 mM Tris-HCl pH 7.7, 20 mM KCl, 10 mM MgCl_2_, 2 mM ATP). The complex was assembled by mixing the individual protein subunits at room temperature in reaction buffer to a final concentration of 5 μM mHsp60 and 6 μM mHsp10 (molar ratio 1:1.2). The mixture was kept at room temperature at all times to avoid the formation of particle strings observed in some vitrified specimens. After only a few seconds of incubation, 3 μl of the mixture were adsorbed onto glow-discharged gold-copper homemade Lacey holey grids and immediately one-side blotted for 4 s from the back of the grid and flash-frozen in liquid ethane using a manual homemade cryoplunger. Overall mixing to vitrification took ~30 s. The grids were imaged on a 300 kV Titan Krios (FEI) equipped with a K2 camera (Gatan) operating in counting mode at a calibrated pixel size of 1.07 Å/pix. We recorded a single movie per hole with a maximum total accumulated exposure of 63 e − /Å^2^ fractionated into 50 frames of 200 ms (yielding movies of 10 s duration). We employed a defocus range between 0.8 and 2.5 μm. Coma-free alignment of the microscope was performed using Leginon^58^. In total, 1883 movies were recorded automatically with Leginon^59^ to control both the microscope and the K2 camera.

### Image processing and EM structure determination

For data processing, frame alignment of each movie was carried out using MotionCor2^60^ and calculated their contrast transfer function (CTF) values with CTFFIND4^61^. The effective defocus calculated by this method ranged from 0.5 to 2.5 mm for the whole data set. The best 1634 movies according to CTF values and total motion were chosen for particle selection. We used FindEM^62^ integrated into the Appion pipeline^63^, to automatically select 162,669 particles using templates generated from low resolution 3D reconstructions of mHsp60–mHsp10 football complexes. 2D and 3D classification and refinements were performed using RELION^64^ without imposing symmetry. The X-ray structure of the mHsp60^E321K^-mHsp10 football complex (PDB 4PJ1) filtered at 60 Å was used as initial model for 3D classification. An initial refinement without imposing symmetry of a subset of 52,261 football particles generated a 4.2 Å resolution 3D reconstruction (based on the gold-standard FSC = 0.143 criterion^30^) with a high degree of D7 symmetry. The data were then transferred to cryoSPARC^65^ and analyzed independently by 2D classification, Ab-initio modeling, and homogeneous refinement. The analysis by cryoSPARC without imposing symmetry generated an initial map at a 3.74 Å resolution based on the gold-standard FSC = 0.143 criterion^30^ from 66,013 particles. Consistent with the observation of a high degree of D7 symmetry in all the reconstructions of the football particle generated both by RELION and cryoSPARC, we decided to impose this symmetry during the refinement yielding a 3D reconstruction of the football complex at 3.08 Å resolution. A smaller subset of images of the half-football complex, consisting in 10,972 particles, generated a C7 symmetry 3.83 Å map of the half-football complex. The local resolution of the 3D reconstructions was determined using ResMap^66^. For model building, we began by rigid-body fitting into our map a single mHsp60 (subunit A) and mHsp10 (subunit O) subunit from the 3.15 Å crystal structure of the ADP-bound mHsp60^E321K^–mHsp10 football complex (PDB 4PJ1)^28^. To improve the fit and to optimize stereochemistry, we carried out real space refinement with secondary structure and geometry restraints and simulated annealing using Phenix^67^.

### Refolding assays

Denaturation of 0.45 µM pig heart MDH was performed in the presence of 5 mM HCl at room temperature. After 1-h incubation, the activity of MDH was measured to confirm the loss of activity. Denatured MDH was diluted into buffer (50 mM Na-HEPES pH 7.4, 50 mM KCl, 20 mM MgCl_2_, and 5 mM DTT) containing mHsp60. After 30 min of incubation at 30 °C, in which the mHsp60–substrate binary complex is formed, the mHsp60-assisted MDH refolding was initiated by adding mHsp10 and ATP. The final monomer concentrations were: 10 µM mHsp60, 20 µM mHsp10, and 0.45 µM MDH (Roche). Following 1 h incubation at room temperature, 20 µl aliquots were taken and assayed for MDH activity by adding 980 µl reaction mixture, which contained 150 mM potassium phosphate pH 7.5, 5 mM DTT, 0.5 mM oxaloacetate, and 0.28 mM NADH. MDH activity was determined by monitoring NADH oxidation as a function of time at 340 nm. The refolding activity is presented relative to the refolding activity of native MDH (100%).

CS (25 μM) was denatured at room temperature in a buffer of 6 M Gdn-HCl, 50 mM Tris-HCl PH 8, 3 mM DTT, and 2 mM EDTA. Then, CS was promptly diluted 100-fold into buffer D (0.15 M Tris-HCl pH 8, 16 mM MgCl_2_, 32 mM KCl) containing 30 μM mHsp60 at 30 °C for 30 min. Refolding was commenced by adding 20 μl of 120 μM mHsp10 and 3 mM ATP in buffer D to 40 μl of the above mixture. CS activity was assayed by using 0.5 mM oxaloacetate and 0.2 mM acetyl-coA as substrates. The condensing reaction was monitored by binding of Ellman’s reagent (DTNB) to the free SH group of the released CoA. The activity of the refolding CS is given as a percentage relative to a control sample of native CS.

D116/G117-eGFP was acid denatured in 200 ml of 30 mM HCl at a final concentration of 12.5 µM and incubated at 25 °C for 1 h. The denatured protein was then diluted 100-fold in 200 ml of 50 mM (3-(N-morpholino)propanesulfonic acid) buffer (pH 7.0) containing 100 mM KCl, 10 mM Mg(CH3COO)_2_, 5 mM DTT, and 0.0125% Tween 20 (refolding buffer). The refolding buffer contained 30 µM mHsp60 with 60 µM mHsp10, folding was initiated by adding different concentrations of ATP. Folding was monitored at 37 °C using a Synergy HT plate reader (Bio Tek) with a 485/20 excitation filter, a 528/20 emission filter, and a gain of 55. The fluorescence recovery is presented relative to the fluorescence of native D116/G117-eGFP (100%).

### Native gel analysis

WT and variant mHsp60 (15 μM) were incubated for 5 min in buffer E (20 mM Tris-HCl pH 7.7, 20 mM MgCl_2_, 50 mM KCl) in the presence or absence of nucleotide, and with and without 1 mM DTT. There was no additional nucleotide in the loading buffer, gel or running buffer. Hence, in this experiment nucleotide is effectively depleted during electrophoresis due to separation from the protein pool and hydrolysis, and not resupplied. We loaded 5 μg of each sample into a 6% native-PAGE.

### SEC-MALS analysis

Samples were incubated at the specified solution conditions, described in the legend to Supplementary Fig. 7. After incubation for 5 min at room temperature (∼25 °C), the proteins were injected into a Superose-6 SEC column (GE Healthcare) that was equilibrated with the same buffer. The column was connected to a MALS detector (DAWN HELEOS II; Wyatt Technology) and then to a refractive index detector (Optilab t-rEX; Wyatt Technology). Wyatt Astra V software was used for data collection and analysis. Data were collected at ∼25 °C.

### In vivo complementation assay

Using the IPTG-inducible pOFX plasmid expressing wild-type human mHsp10 and mHsp60^29^, we engineered two additional constructs containing human mHsp10 and the single-ring mHsp60 mutants. The mutations S464R and S464R/K109E were inserted into pOFX by Gibson assembly and transformed into *E. coli* NEB5α. Following purification from NEB5α, the pOFX plasmids were transformed by electroporation into MGM100 competent cells. Finally, the ability of cells were grown on 2YT-agar plates containing 25 mg/ml kanamycin and 50 mg/ml spectinomycin in the presence of either: 0.2% arabinose, 0.5% glucose or 0.5% glucose, and 1 mM IPTG.

### Reporting summary

Further information on research design is available in the Nature Research Reporting Summary linked to this article.

## Supplementary information

Supplementary Information

Reporting Summary

## Data Availability

The EM maps of ADP-bound mHsp60–mHsp10 football and half-football complexes have been deposited in the Electron Microscopy Data Bank (http://www.ebi.ac.uk/pdbe/emdb/) under accession numbers EMD-9195 and EMD-9196, respectively. The atomic coordinates of ADP-bound mHsp60–mHsp10 football, ADP:BeF_3_ bound mHsp60–mHsp10 football and ADP-bound half-football complexes have been deposited in the Protein Data Bank (www.pdb.org) under PDB 6MRC, PDB 6HT7, and PDB 6MRD, respectively. The source data underlying Figs. 5e, 6a–d and Supplementary Figs 7a–c are provided as a Source Data file. All reagents and relevant data are available from the corresponding authors upon reasonable request.

## Source Data

Source Data

## References

[1] Levy-Rimler G, Bell RE, Ben-Tal N, Azem A (2002). Type I chaperonins: not all are created equal. FEBS Lett..

[2] Cheng MY (1989). Mitochondrial heat-shock protein hsp60 is essential for assembly of proteins imported into yeast mitochondria. Nature.

[3] Christensen JH (2010). Inactivation of the hereditary spastic paraplegia-associated Hspd1 gene encoding the Hsp60 chaperone results in early embryonic lethality in mice. Cell Stress Chaperones.

[4] Cappello F, Conway de Macario E, Marasa L, Zummo G, Macario AJ (2008). Hsp60 expression, new locations, functions and perspectives for cancer diagnosis and therapy. Cancer Biol. Ther..

[5] Osterloh A (2004). Lipopolysaccharide-free heat shock protein 60 activates T cells. J. Biol. Chem..

[6] Johnson BJ (2005). Heat shock protein 10 inhibits lipopolysaccharide-induced inflammatory mediator production. J. Biol. Chem..

[7] Xanthoudakis S (1999). Hsp60 accelerates the maturation of pro-caspase-3 by upstream activator proteases during apoptosis. EMBO J..

[8] Knowlton AA, Gupta S (2003). HSP60, Bax, and cardiac apoptosis. Cardiovas. Tox.

[9] Ban HS, Shimizu K, Minegishi H, Nakamura H (2010). Identification of HSP60 as a primary target of o-carboranylphenoxyacetanilide, an HIF-1alpha inhibitor. J. Am. Chem. Soc..

[10] Chun JN (2010). Cytosolic Hsp60 is involved in the NF-kappaB-dependent survival of cancer cells via IKK regulation. PLoS ONE.

[11] Hansen J (2007). A novel mutation in the HSPD1 gene in a patient with hereditary spastic paraplegia. J. Neurol..

[12] Hansen JJ (2002). Hereditary spastic paraplegia SPG13 is associated with a mutation in the gene encoding the mitochondrial chaperonin Hsp60. Am. J. Hum. Genet..

[13] Magen D (2008). Mitochondrial hsp60 chaperonopathy causes an autosomal-recessive neurodegenerative disorder linked to brain hypomyelination and leukodystrophy. Am. J. Hum. Genet..

[14] Briones P (1997). A new case of multiple mitochondrial enzyme deficiencies with decreased amount of heat shock protein 60. J. Inherit. Metab. Dis..

[15] Venner TJ, Gupta RS (1990). Nucleotide sequence of mouse HSP60 (chaperonin, GroEL homolog) cDNA. Biochim Biophys. Acta.

[16] Venner TJ, Singh B, Gupta RS (1990). Nucleotide sequences and novel structural features of human and Chinese hamster hsp60 (chaperonin) gene families. DNA Cell Biol..

[17] Rye HS (1997). Distinct actions of cis and trans ATP within the double ring of the chaperonin GroEL. Nature.

[18] Xu Z, Horwich AL, Sigler PB (1997). The crystal structure of the asymmetric GroEL-GroES-(ADP)7 chaperonin complex. Nature.

[19] Horovitz A, Fridmann Y, Kafri G, Yifrach O (2001). Review: allostery in chaperonins. J. Struct. Biol..

[20] Saibil HR, Fenton WA, Clare DK, Horwich AL (2013). Structure and allostery of the chaperonin GroEL. J. Mol. Biol..

[21] Levy-Rimler G (2001). The effect of nucleotides and mitochondrial chaperonin 10 on the structure and chaperone activity of mitochondrial chaperonin 60. Eur. J. Biochem./FEBS.

[22] Yifrach O, Horovitz A (1995). Nested cooperativity in the ATPase activity of the oligomeric chaperonin GroEL. Biochemistry.

[23] Sameshima T, Iizuka R, Ueno T, Funatsu T (2010). Denatured proteins facilitate the formation of the football-shaped GroEL-(GroES)2 complex. Biochem. J..

[24] Ye X, Lorimer GH (2013). Substrate protein switches GroE chaperonins from asymmetric to symmetric cycling by catalyzing nucleotide exchange. Proc. Natl Acad. Sci. USA.

[25] Yang D, Ye X, Lorimer GH (2013). Symmetric GroEL:GroES2 complexes are the protein-folding functional form of the chaperonin nanomachine. Proc. Natl Acad. Sci. USA.

[26] Nielsen KL, Cowan NJ (1998). A single ring is sufficient for productive chaperonin-mediated folding in vivo. Mol. Cell.

[27] Weiss C, Jebara F, Nisemblat S, Azem A (2016). Dynamic Complexes in the Chaperonin-Mediated Protein Folding Cycle. Front. Mol. Biosci..

[28] Nisemblat S, Yaniv O, Parnas A, Frolow F, Azem A (2015). Crystal structure of the human mitochondrial chaperonin symmetrical football complex. Proc. Natl Acad. Sci. USA.

[29] Parnas A (2012). Identification of elements that dictate the specificity of mitochondrial Hsp60 for its co-chaperonin. PLoS ONE.

[30] Scheres SH, Chen S (2012). Prevention of overfitting in cryo-EM structure determination. Nat. Methods.

[31] Hunt JF, Weaver AJ, Landry SJ, Gierasch L, Deisenhofer J (1996). The crystal structure of the GroES co-chaperonin at 2.8 A resolution. Nature.

[32] Parnas A (2009). The MitCHAP-60 disease is due to entropic destabilization of the human mitochondrial Hsp60 oligomer. J. Biol. Chem..

[33] Ma J, Karplus M (1998). The allosteric mechanism of the chaperonin GroEL: a dynamic analysis. Proc. Natl Acad. Sci. USA.

[34] Braig K (1994). The crystal structure of the bacterial chaperonin GroEL at 2.8 A. Nature.

[35] Yifrach O, Horovitz A (1996). Allosteric control by ATP of non-folded protein binding to GroEL. J. Mol. Biol..

[36] Fei X, Yang D, LaRonde-LeBlanc N, Lorimer GH (2013). Crystal structure of a GroEL-ADP complex in the relaxed allosteric state at 2.7 A resolution. Proc. Natl Acad. Sci. USA.

[37] Fei X, Ye X, LaRonde NA, Lorimer GH (2014). Formation and structures of GroEL:GroES2 chaperonin footballs, the protein-folding functional form. Proc. Natl Acad. Sci. USA.

[38] Gruber R, Levitt M, Horovitz A (2017). Sequential allosteric mechanism of ATP hydrolysis by the CCT/TRiC chaperone is revealed through Arrhenius analysis. Proc. Natl Acad. Sci. USA.

[39] Lorimer, G. H., Fei, X. & Ye, X. The GroEL chaperonin: a protein machine with pistons driven by ATP binding and hydrolysis. *Philos. Trans. R Soc. Lond. B Biol. Sci.* **373**, 10.1098/rstb.2017.0179 (2018). 10.1098/rstb.2017.0179 PMC5941174 29735733

[40] Todd MJ, Viitanen PV, Lorimer GH (1994). Dynamics of the chaperonin ATPase cycle: implications for facilitated protein folding. Science.

[41] Sewell BT (2004). A mutant chaperonin with rearranged inter-ring electrostatic contacts and temperature-sensitive dissociation. Nat. Struct. Mol. Biol..

[42] Sot B (2005). Ionic interactions at both inter-ring contact sites of GroEL are involved in transmission of the allosteric signal: a time-resolved infrared difference study. Protein Sci..

[43] Bandyopadhyay B, Mondal T, Unger R, Horovitz A (2019). Contact order is a determinant for the dependence of GFP folding on the chaperonin GroEL. Biophys. J..

[44] Viitanen PV (1998). Purification of mammalian mitochondrial chaperonin 60 through in vitro reconstitution of active oligomers. Methods Enzymol..

[45] Horwich AL, Fenton WA (2009). Chaperonin-mediated protein folding: using a central cavity to kinetically assist polypeptide chain folding. Q Rev. Biophys..

[46] Koike-Takeshita A, Arakawa T, Taguchi H, Shimamura T (2014). Crystal structure of a symmetric football-shaped GroEL:GroES2-ATP14 complex determined at 3.8A reveals rearrangement between two GroEL rings. J. Mol. Biol..

[47] Goodsell DS, Olson AJ (2000). Structural symmetry and protein function. Annu. Rev. Biophys. Biomol. Struct..

[48] Andre I, Strauss CE, Kaplan DB, Bradley P, Baker D (2008). Emergence of symmetry in homooligomeric biological assemblies. Proc. Natl Acad. Sci. USA.

[49] Yan X (2018). GroEL ring separation and exchange in the chaperonin reaction. Cell.

[50] Farr GW (2003). Folding with and without encapsulation by cis- and trans-only GroEL-GroES complexes. EMBO J..

[51] Bigman LS, Horovitz A (2019). Reconciling the controversy regarding the functional importance of bullet- and football-shaped GroE complexes. J. Biol. Chem..

[52] Opatowsky Y, Chomsky-Hecht O, Kang MG, Campbell KP, Hirsch JA (2003). The voltage-dependent calcium channel beta subunit contains two stable interacting domains. J. Biol. Chem..

[53] Goloubinoff P, Diamant S, Weiss C, Azem A (1997). GroES binding regulates GroEL chaperonin activity under heat shock. FEBS Lett..

[54] Sokolovski M, Bhattacherjee A, Kessler N, Levy Y, Horovitz A (2015). Thermodynamic protein destabilization by GFP tagging: a case of interdomain allostery. Biophysical J..

[55] Kabsch W (2010). Xds. Acta Crystallogr D. Biol. Crystallogr.

[56] Afonine PV (2012). Towards automated crystallographic structure refinement with phenix.refine. Acta Crystallogr. D. Biol. Crystallogr..

[57] Emsley P, Cowtan K (2004). Coot: model-building tools for molecular graphics. Acta Crystallogr. D. Biol. Crystallogr..

[58] Glaeser RM, Typke D, Tiemeijer PC, Pulokas J, Cheng A (2011). Precise beam-tilt alignment and collimation are required to minimize the phase error associated with coma in high-resolution cryo-EM. J. Struct. Biol..

[59] Suloway C (2005). Automated molecular microscopy: the new Leginon system. J. Struct. Biol..

[60] Zheng SQ (2017). MotionCor2: anisotropic correction of beam-induced motion for improved cryo-electron microscopy. Nat. Methods.

[61] Rohou A, Grigorieff N (2015). CTFFIND4: fast and accurate defocus estimation from electron micrographs. J. Struct. Biol..

[62] Roseman AM (2004). FindEM—a fast, efficient program for automatic selection of particles from electron micrographs. J. Struct. Biol..

[63] Lander GC (2009). Appion: an integrated, database-driven pipeline to facilitate EM image processing. J. Struct. Biol..

[64] Scheres SH (2012). RELION: implementation of a Bayesian approach to cryo-EM structure determination. J. Struct. Biol..

[65] Punjani A, Rubinstein JL, Fleet DJ, Brubaker MA (2017). cryoSPARC: algorithms for rapid unsupervised cryo-EM structure determination. Nat. Methods.

[66] Kucukelbir A, Sigworth FJ, Tagare HD (2014). Quantifying the local resolution of cryo-EM density maps. Nat. Methods.

[67] Adams PD (2010). PHENIX: a comprehensive Python-based system for macromolecular structure solution. Acta Crystallogr. D. Biol. Crystallogr..

