# Supplementary material for: Structural basis for active single and double ring complexes in human mitochondrial Hsp60-Hsp10 chaperonin
Source: Nat Commun. 2020 Apr 21;11:1916. doi: 10.1038/s41467-020-15698-8 (PMC7174398; doi:10.1038/s41467-020-15698-8)
Supplement: Supplementary file 1 — Supplementary Information [file 41467_2020_15698_MOESM1_ESM.pdf]

a

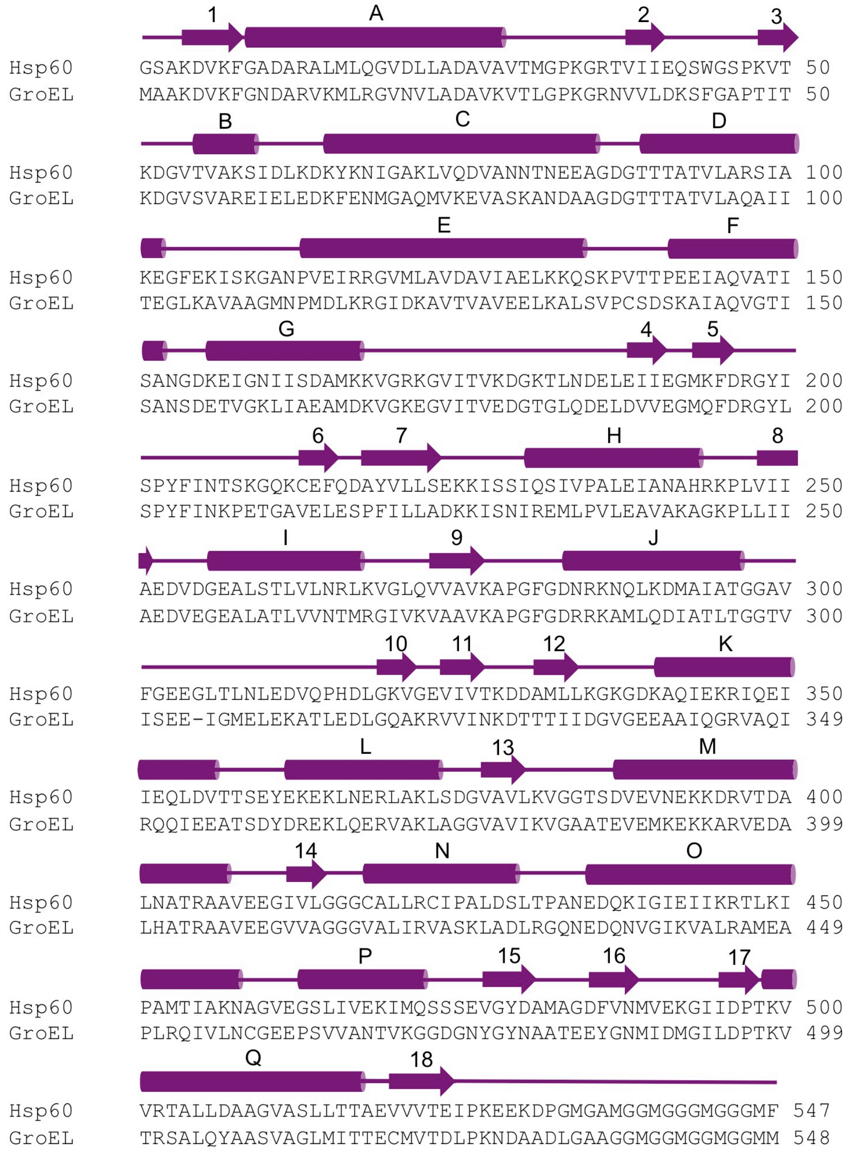

b

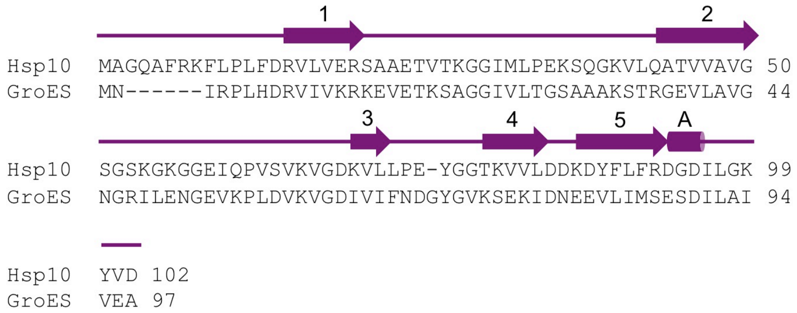

Supplementary Figure 1. a. Sequence alignment between human mHsp60 and GroEL (wt mHsp60 exhibits 51% sequence identity with GroEL). **b.** Sequence alignment between human mHsp10 and GroES (wt mHsp10 exhibits 33% sequence identity with GroES). In both panels, numbered arrows indicate β-sheets, and alphabetically marked cylinders represent α-helices.

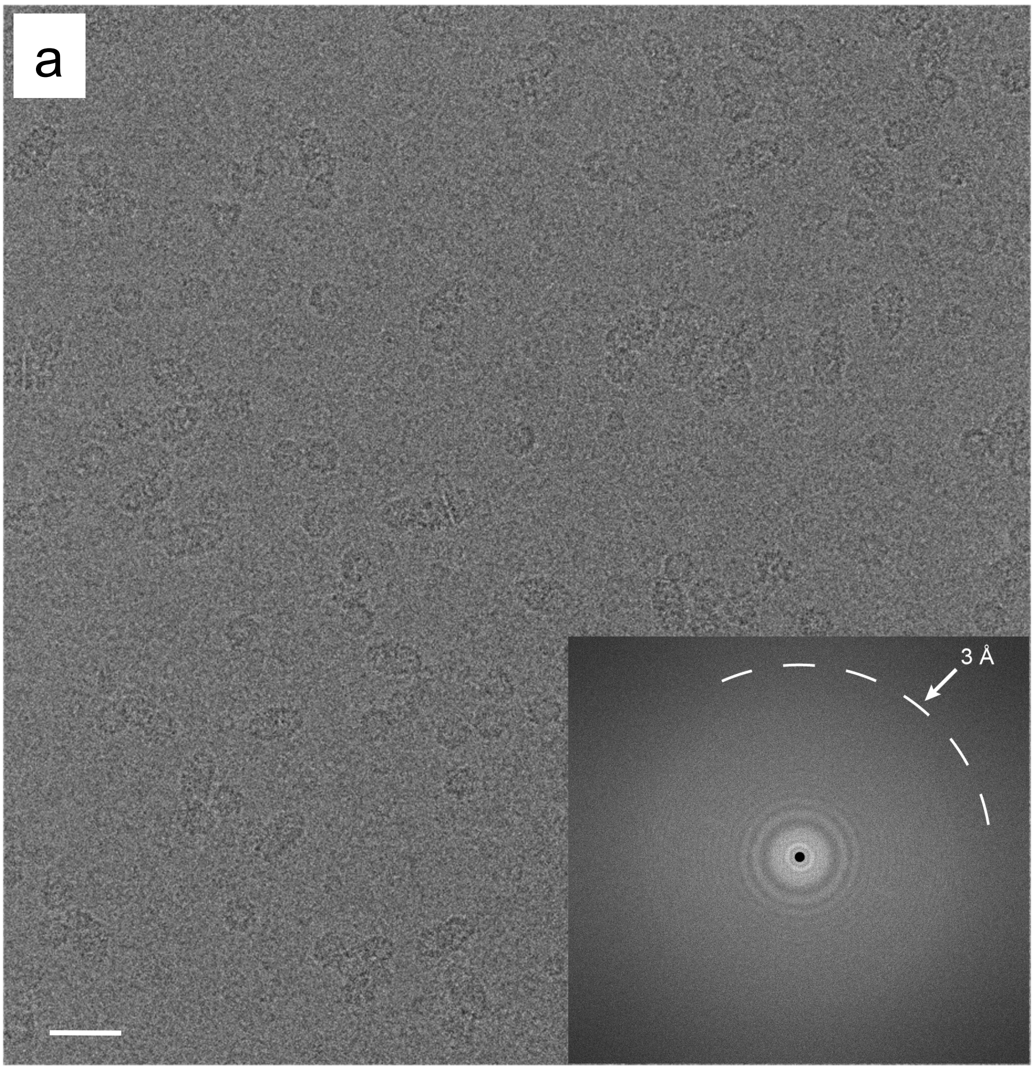

b

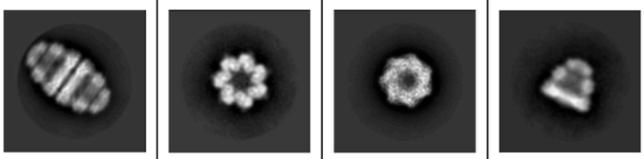

sample	inc. t	"football" side view ($mHsp60_{14}\cdot mHsp10_{14}$)	"football" front view ($mHsp60_{14}\cdot mHsp10_{14}$)	half-football front view ($mHsp60_{7}\cdot mHsp10_{7}$)	half-football side view ($mHsp60_{7}\cdot mHsp10_{7}$)	half-footb. ratio (side view)
mHsp60·mHsp10, ATP	30 sec	51k (62%)	4.0k (5%)	11k (14%)	12k (15%)	4.2
mHsp60·mHsp10, ATP	5 min	13k (70%)	1.1k (6%)	1.6k (9%)	3.0k (16%)	4.3
mHsp60·mHsp10, ATP	30 min	16k (63%)	3.2k (12%)	2.0k (8%)	4.5 (17%)	3.5

Supplementary Figure 2. a. Electron micrograph (aligned movie) of vitrified mHsp60-mHsp10 complexes. The scale bar represents 250 Å. The inset displays the Fourier transform of the micrograph with Thon rings extending to 3 Å. **b.** Distribution of particle types as a function of incubation time.

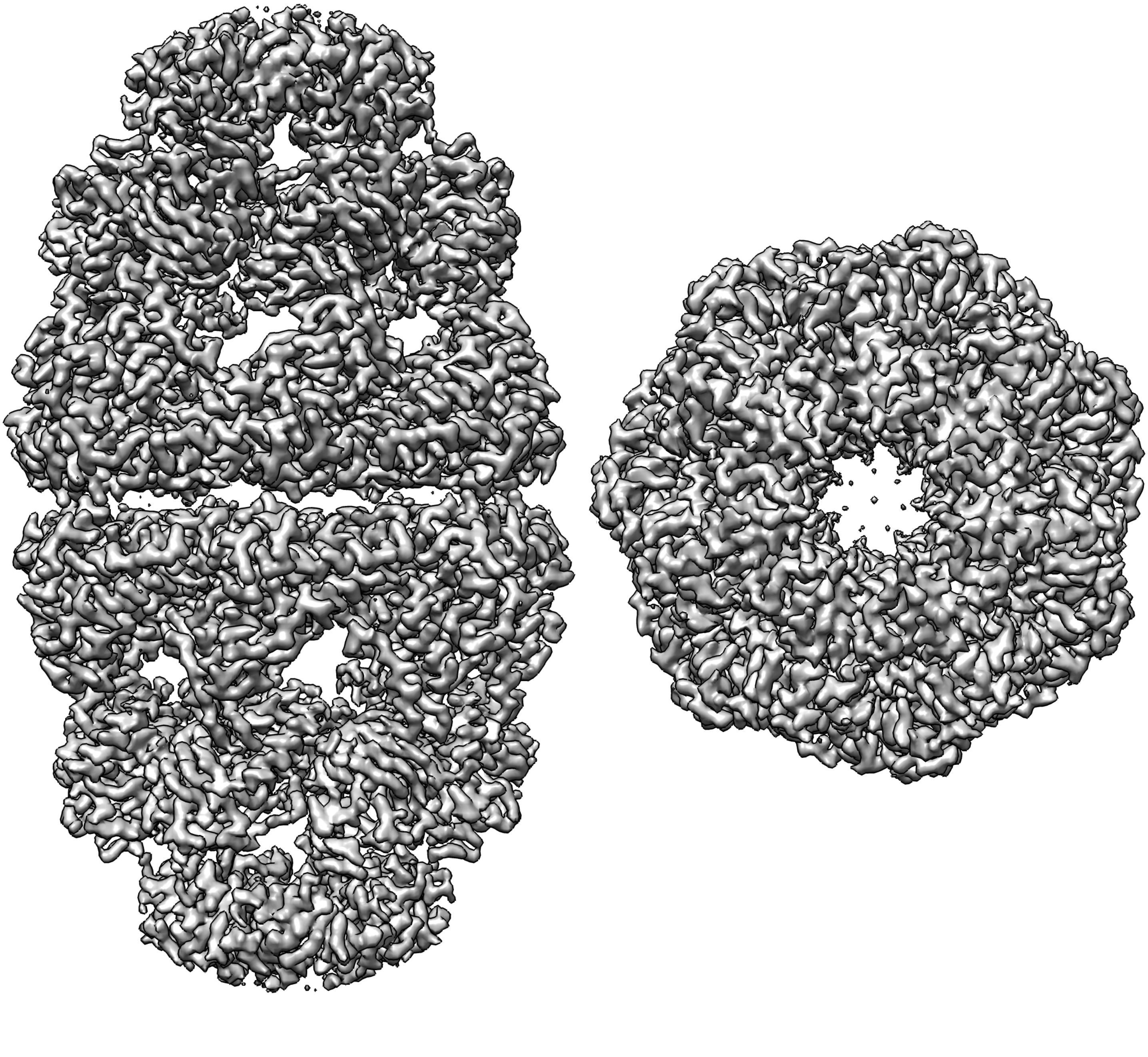

Supplementary Figure 3. Side and top views of the mHsp60$_{14}$-(mHsp10$_7$)$_2$ ADP football 3.08 Å D7-symmetrized cryo-EM map.

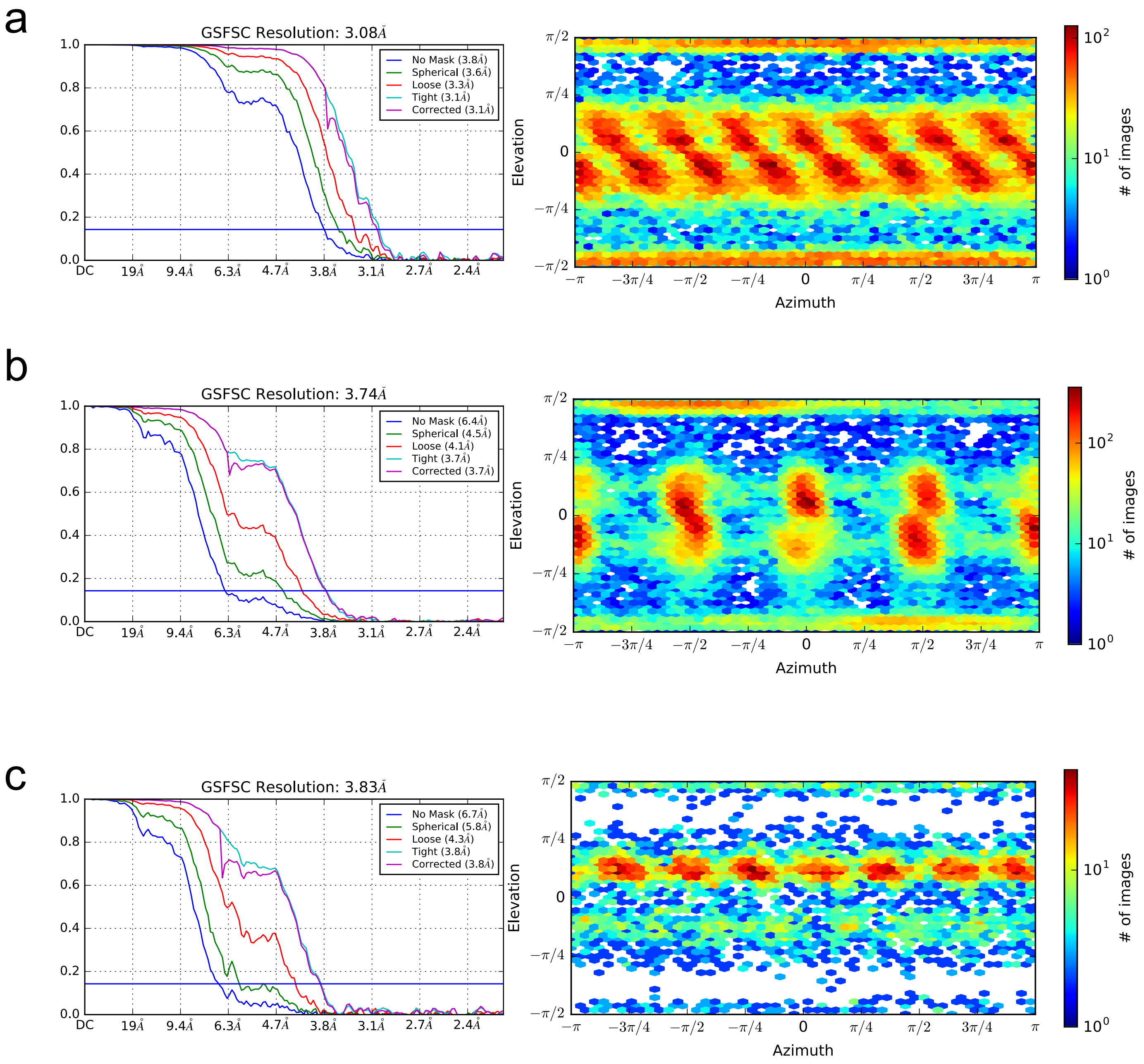

Supplementary Figure 4. a. Left: cryoSPARC-generated FSC curve for the final 3-D reconstruction of the D7-symmetrized mHsp60$_{14}$-(mHsp10$_7$)$_2$ ADP football complex. Right: cryoSPARC-generated viewing direction distribution plot for the final 3-D reconstruction of the same. **b.** Left: cryoSPARCgenerated FSC curve for the final 3-D reconstruction of the C1-symmetrized mHsp60$_{14}$-(mHsp10$_7$)$_2$ ADP football complex. Right: cryoSPARC-generated viewing direction distribution plot for the final 3-D reconstruction of the same. **c.** Left: cryoSPARC-generated FSC curve for the final 3-D reconstruction of the C7-symmetrized mHsp60$_7$-mHsp10$_7$ ADP half-football complex. Right: cryoSPARC-generated viewing direction distribution plot for the same.

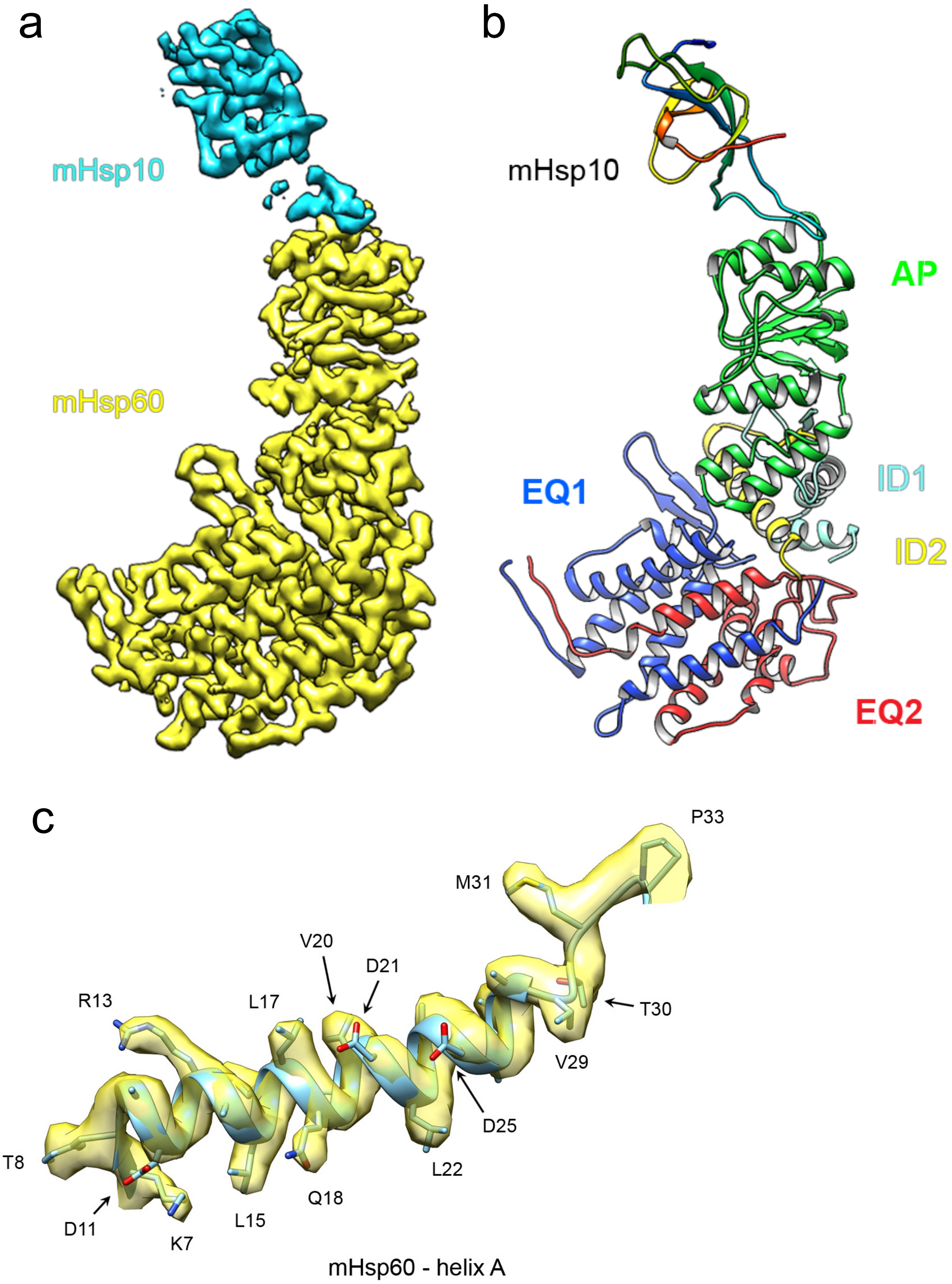

Supplementary Figure 5. **a.** Region of ADP bound mHsp60$_{14}$-(mHsp10$_7$)$_2$ ADP cryo-EM map corresponding to one mHsp60/mHsp10 dimer. mHsp60 is colored in yellow and mHsp10 in blue. **b.** Ribbon diagram of the atomic model of the mHsp60/mHsp10 dimer calculated from the cryo-EM density map. The different domains of mHsp60 are indicated: equatorial 1 (EQ1), equatorial 2 (EQ2), intermediate 1 (ID1), intermediate 2 (ID2) and apical (AP). **c.** Detailed view of mHsp60 helix A of the D7-symmetrized 3.08 Å ADP football cryo-EM map. The atomic model is shown inside the EM density (yellow) and side chains of the corresponding residues are labeled

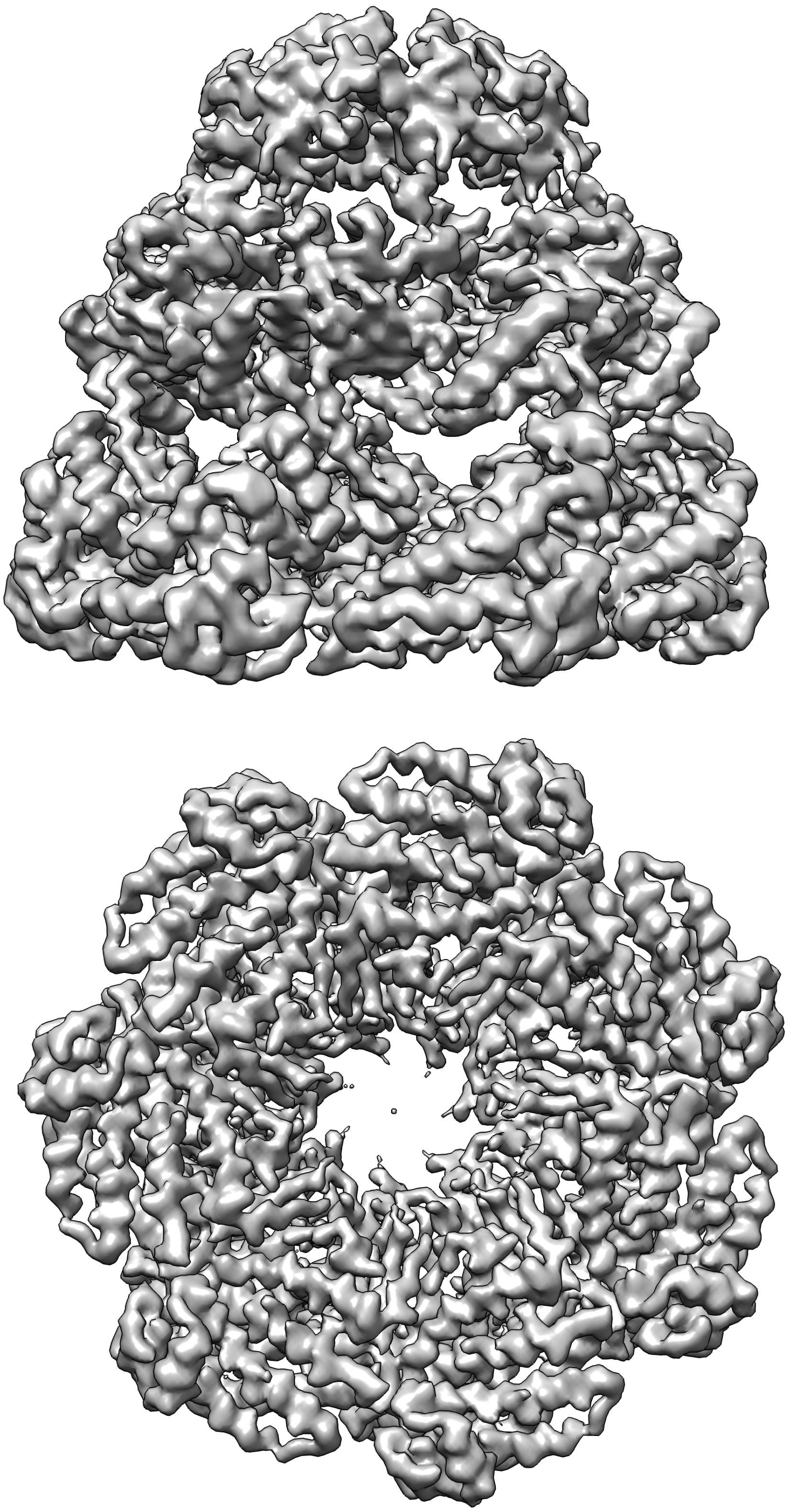

Supplementary Figure 6. Side and top views of the $mHsp60_7$-$mHsp10_7$ ADP football 3.83 Å C7-symmetrized cryo-EM map.

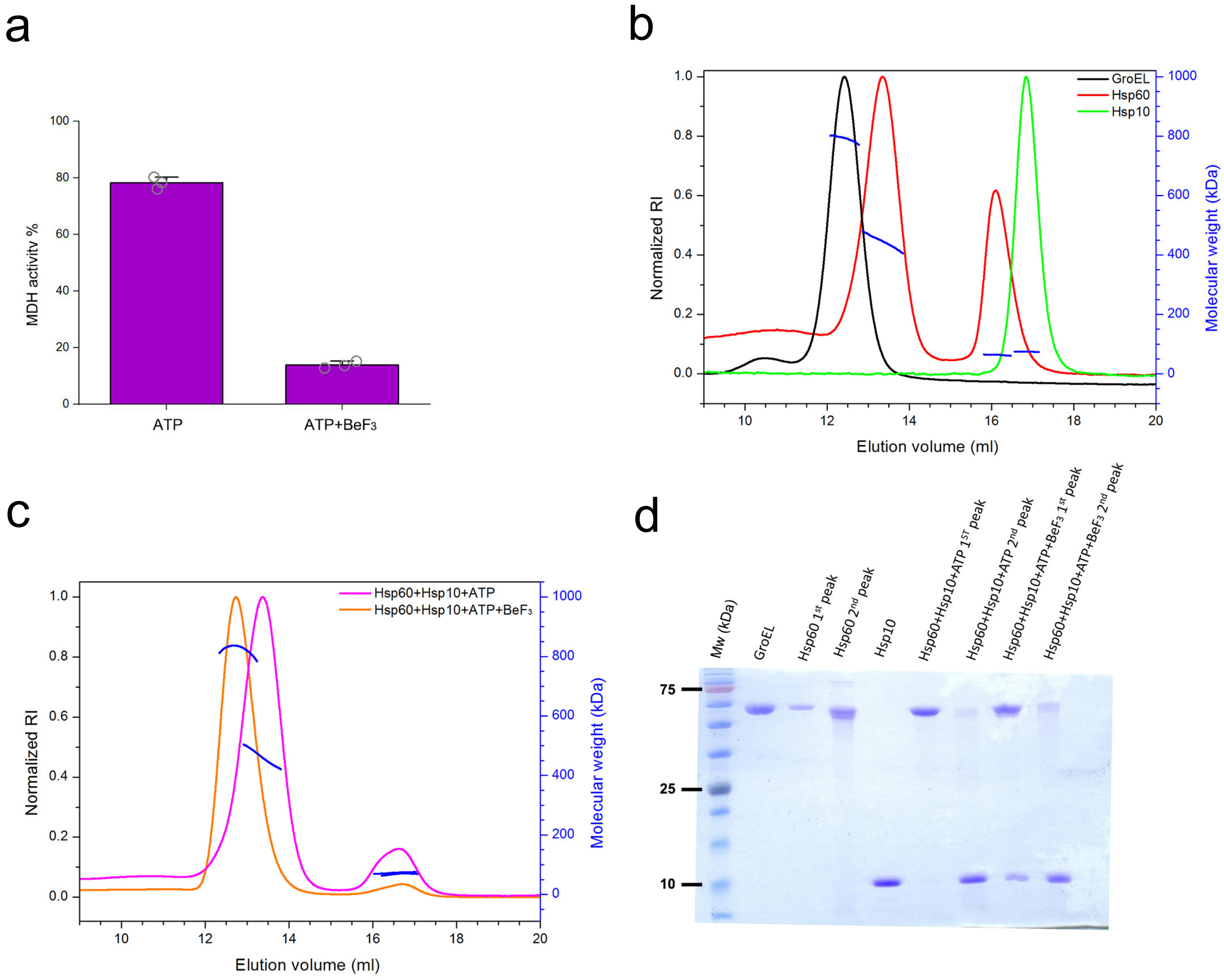

Supplementary Figure 7. a. In vitro folding assay for the mHsp60-mHsp10 system in the presence of ATP or ATP: BeF3. Data represent average ± s.d. from three independent experiments (n=3). Source data are provided as a Source Data file. **b.** SEC-MALS profiles in the absence of ATP of GroEL (black, apparent molecular weight: 792 kDa), wt mHsp60 (red, apparent molecular weight of first and second peaks are 445 and64 kDa, respectively) injected at a concentration of 150 μM, and wt mHsp10 (green, apparent molecular weight: 75 kDa) injected at a concentration of 300 μM. **c.** SEC-MALS profiles of wt mHsp60-mHsp10 complexes. For the mHsp60-mHsp10 complexes, mHsp60 (150 μM) and mHsp10 (300 μM) were incubated for 5 min with 1 mM ATP (magenta, apparent molecular weight of first and second peaks are 462 and 73 kDa, respectively) or 1 mM ATP, 1 mM BeCl2 and 10 mM NaF (orange, apparent molecular weight of first and second peaks are 823 and 68 kDa, respectively). The SEC profiles are plotted against the normalized refractive index (RI) and the measured molecular weight (blue lines) and were run on an analytical Superose-6 size-exclusion column (GE Healthcare). Source data are provided as a Source Data file. **d.** SDS-PAGE of eluted protein peaks from panels **(b, c)**. The gels are representative of at least two independent experiments.

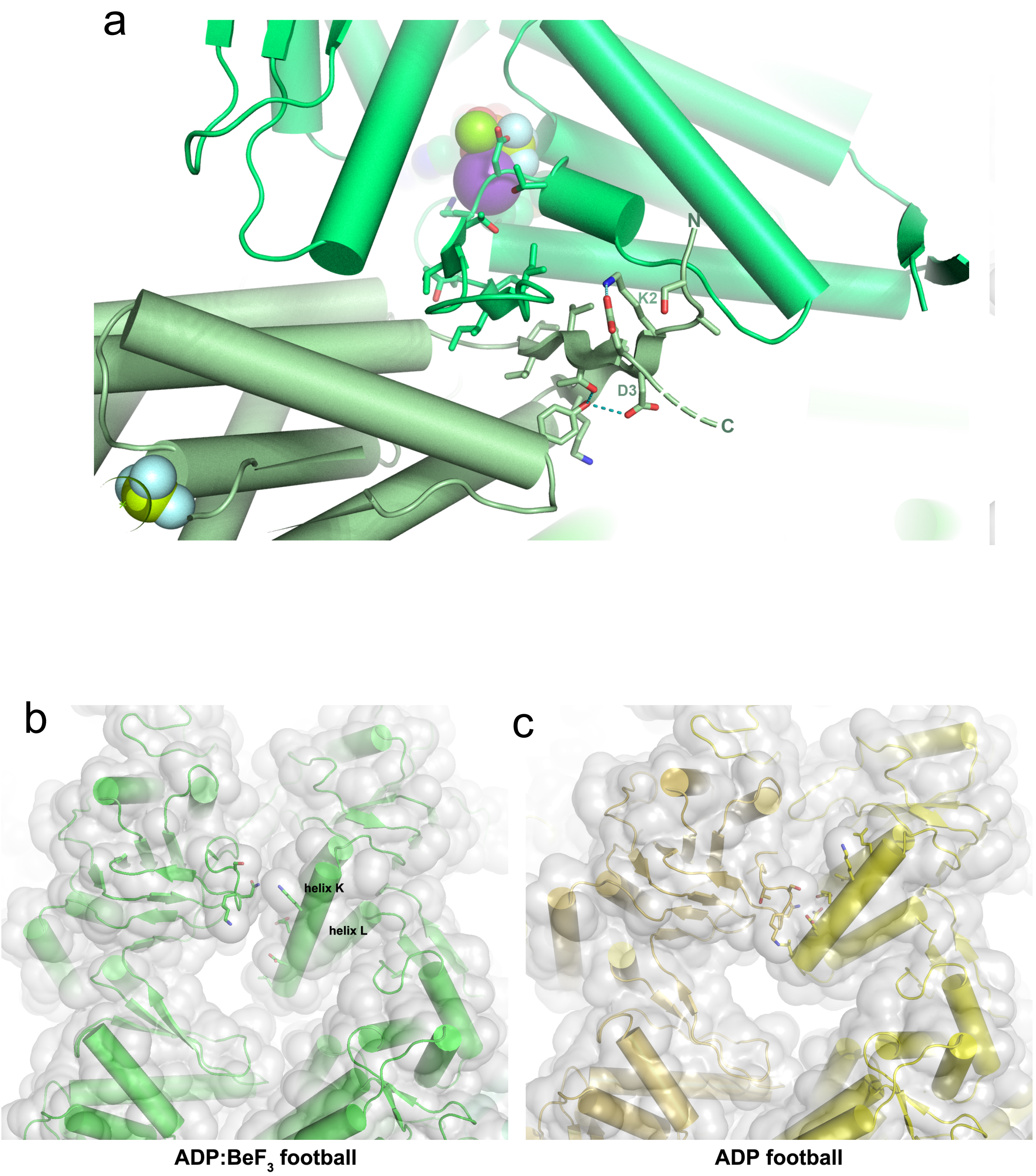

Supplementary Figure 8. a. A view of the equatorial domain β-sheet comprising an intersubunit interface in the ADP:BeF_3 football. Individual subunits are colored in different shades of green. The proximity of the β-hairpin to the BeF_3, Mg^{2+} (green) and K^+ (purple) moieties from the nucleotide is readily apparent, so that ATP or its mimic will position the hairpin to interact with two β-strands of the neighboring subunit. Hydrogen bonds are depicted as blue dots. **(b, c)** Interactions in the apical and intermediate domains for the ADP: BeF_3 football (green) and the ADP football (yellow), respectively labeled. Note how the neighboring subunit loop (left) slides along a face of helix K in the conformational transition from ground state to ADP hydrolysis.

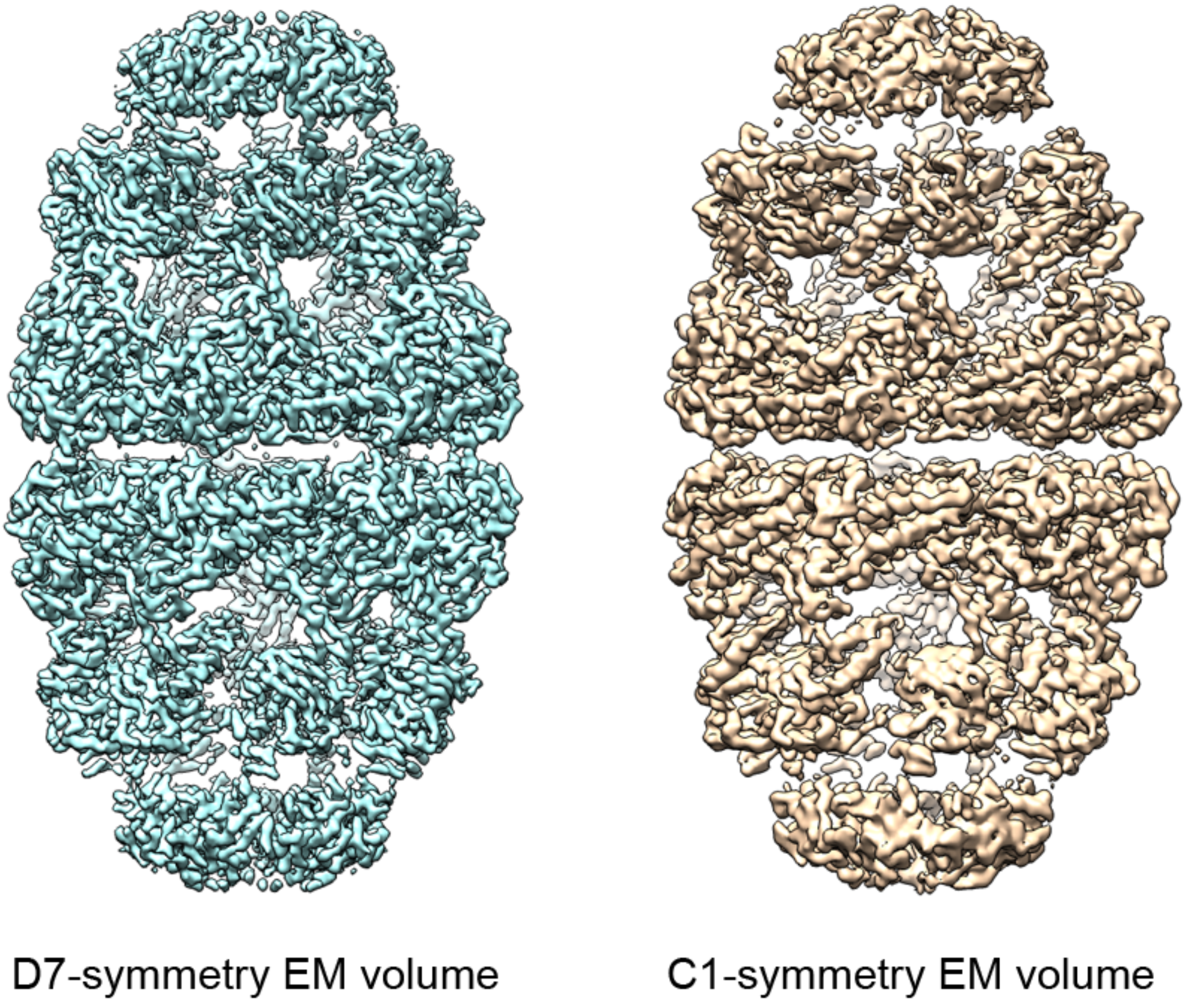

Supplementary Figure 9. Subunit symmetry of the mHsp60-mHsp10 ADP football complexes. 3-D reconstruction of the ADP bound $mHsp60_{14}$-$(mHsp10_7)_2$ football imposing D7 symmetry during refinement (left), and without symmetry imposition (right, C1 symmetry). The resolution for both cryo-EM maps is indicated.

Supplementary Table 1. Superposition RMSD values (Å) between mHsp10 subunits within the ground state (ADP:BeF$_3$) football complex

Sub	O	P	Q	R	S	T	U	V	W	X	Y	Z	1
**P**	0.18												
**Q**	0.2	0.2											
**R**	0.21	0.17	0.18										
**S**	0.24	0.19	0.2	0.17									
**T**	0.24	0.22	0.2	0.18	0.18								
**U**	0.21	0.22	0.24	0.23	0.24	0.26							
**V**	0.16	0.13	0.2	0.18	0.21	0.21	0.25						
**W**	0.23	0.23	0.13	0.19	0.19	0.18	0.27	0.22					
**X**	0.17	0.17	0.24	0.19	0.23	0.24	0.18	0.19	0.27				
**Y**	0.16	0.18	0.18	0.21	0.21	0.22	0.2	0.17	0.22	0.22			
**Z**	0.15	0.16	0.2	0.19	0.21	0.22	0.21	0.16	0.24	0.19	0.16		
**1**	0.19	0.18	0.21	0.17	0.19	0.22	0.22	0.19	0.22	0.19	0.21	0.18	
**2**	0.14	0.15	0.2	0.19	0.2	0.23	0.21	0.15	0.24	0.18	0.15	0.13	0.16

Supplementary Table 2. Superposition RMSD values (Å) between mHsp10 subunits in the ground state (ADP:BeF_3) football complex and respective ADP complexes

ADP:BeF_3 football complex	ADP football	ADP half-football
**O**	0.19	0.49
**P**	0.18	0.5
**Q**	0.2	0.5
**R**	0.17	0.45
**S**	0.2	0.45
**T**	0.21	0.46
**U**	0.22	0.49
**V**	0.17	0.5
**W**	0.23	0.49
**X**	0.21	0.5
**Y**	0.17	0.47
**Z**	0.17	0.48
**1**	0.17	0.47
**2**	0.16	0.49

*The RMSD value between Q subunits from ADP football and ADP half-football is 0.44 Å.

Supplementary Table 3. Superposition RMSD values (Å) between mHsp60 subunits in the ground state (ADP:BeF_3) football complex

Subunit	A	B	C	D	E	F	G	H	I	J	K	L	M
**B**	0.6						0.72						
**C**	1.54	1.59											
**D**	1.74	1.49	0.61										
**E**	1.86	1.6	0.87	0.37									
**F**	1.92/1.88	1.83/1.74	1.34	1.02	0.97								
**G**	0.9	0.72	1.9	1.59	1.8	1.71/1.69							
**H**	0.42	0.64	1.47	1.66	1.72	1.96	1.07						
**I**	1.66/1.56	1.6	0.27	0.51	0.76	1.27	1.86	1.52					
**J**	1.2	0.79	1.87	1.84/1.81	1.96/1.9	2.19	0.72	1.25	1.84				
**K**	0.47	0.79	1.67/1.65	1.7/1.73	1.87/1.89	1.64	1.01	0.56	1.71	1.27			
**L**	0.42	0.6	1.58	1.76	1.77	1.93/1.88	0.99	0.32	1.64	1.15	0.62		
**M**	0.68	0.43	1.69/1.66	1.76	1.8	1.81/1.76	0.56	0.79	1.74	0.67	0.89	0.69	
**N**	0.37	0.54	1.44	1.62	1.84/1.85	1.8	0.98	0.48	1.67	1.15	0.49	0.51	0.69

Supplementary Table 4. Superposition RMSD values (Å) between mHsp60 subunits in the ground state (ADP:BeF_3) football complex and respective ADP complexes

(ADP:BeF_3)	ADP football	ADP half-football
**A**	0.78	0.8
**B**	0.84	0.91
**C**	1.69	1.74
**D**	1.84	1.78
**E**	1.81	1.8
**F**	1.72	1.58
**G**	0.91	1.03
**H**	0.82	0.81
**I**	1.59	1.75
**J**	1.15	1.27
**K**	1.04	0.99
**L**	0.86	0.89
**M**	0.74	0.86
**N**	0.99	0.96

Supplementary Table 5. Buried surface area and volume values

Complex	BSA ($Å^2$)	Volume ($Å^3$)
**ADP:BeF$_3$ football**		
football		988817
north half	6359.8	
south half	6012.3	
ring interface	4237.1	
**ADP football**		
football		962467
north half	6379.2	
south half	6403.0	
ring interface	3200.2	
**4PJ1**		
football		1033747
north half	6833.6	
south half	6521.4	
ring interface	11560.6	
