# Supplementary material for: Structural basis for active single and double ring complexes in human mitochondrial Hsp60-Hsp10 chaperonin
Source: Nat Commun. 2020 Apr 21;11:1916. doi: 10.1038/s41467-020-15698-8 (PMC7174398; doi:10.1038/s41467-020-15698-8)
Supplement: Supplementary file 2 — Reporting Summary [file 41467_2020_15698_MOESM2_ESM.pdf]

nature research

Corresponding author(s): Abdussalam Azem, Joel A. Hirsch, and Iban Ubarretxena-Belandia

Last updated by author(s): Mar 11, 2020

Reporting Summary

Nature Research wishes to improve the reproducibility of the work that we publish. This form provides structure for consistency and transparency in reporting. For further information on Nature Research policies, see Authors & Referees and the Editorial Policy Checklist .

Statistics

For all statistical analyses, confirm that the following items are present in the figure legend, table legend, main text, or Methods section.

n/a	Confirmed	
☐	☒	The exact sample size (*n*) for each experimental group/condition, given as a discrete number and unit of measurement
☐	☒	A statement on whether measurements were taken from distinct samples or whether the same sample was measured repeatedly
☒	☐	The statistical test(s) used AND whether they are one- or two-sided<br>*Only common tests should be described solely by name; describe more complex techniques in the Methods section.*
☒	☐	A description of all covariates tested
☒	☐	A description of any assumptions or corrections, such as tests of normality and adjustment for multiple comparisons
☒	☐	A full description of the statistical parameters including central tendency (e.g. means) or other basic estimates (e.g. regression coefficient) AND variation (e.g. standard deviation) or associated estimates of uncertainty (e.g. confidence intervals)
☒	☐	For null hypothesis testing, the test statistic (e.g. *F*, *t*, *r*) with confidence intervals, effect sizes, degrees of freedom and *P* value noted<br>*Give P values as exact values whenever suitable.*
☒	☐	For Bayesian analysis, information on the choice of priors and Markov chain Monte Carlo settings
☒	☐	For hierarchical and complex designs, identification of the appropriate level for tests and full reporting of outcomes
☒	☐	Estimates of effect sizes (e.g. Cohen's *d*, Pearson's *r*), indicating how they were calculated

Our web collection on statistics for biologists contains articles on many of the points above.

Software and code

Policy information about availability of computer code

Data collection	MXCube2, Leginon
Data analysis	MotionCor2, RELION, cryoSPARC, ResMap, FindEM, CTFFIND4, Chimera, COOT, PHENIX, EMRinger, MolProbity, ClustalOmega and Uniprot.

For manuscripts utilizing custom algorithms or software that are central to the research but not yet described in published literature, software must be made available to editors/reviewers. We strongly encourage code deposition in a community repository (e.g. GitHub). See the Nature Research guidelines for submitting code & software for further information.

Data

Policy information about availability of data

All manuscripts must include a data availability statement. This statement should provide the following information, where applicable:

- Accession codes, unique identifiers, or web links for publicly available datasets
- A list of figures that have associated raw data
- A description of any restrictions on data availability

Cryo-EM maps of ADP bound mHsp60-mHsp10 football and ADP bound half-football have been deposited with the Electron Microscopy Data Bank under accession numbers EMD-9195 and EMD-9196, respectively. The atomic coordinates of ADP:BeF3 bound mHsp60-mHsp10 football, ADP bound mHsp60-mHsp10 football, and ADP bound half-football complexes have been deposited in the Protein Data Bank (www.pdb.org) under PDB ID codes 6HT7, 6MRC and 6MRD, respectively.

Field-specific reporting

Please select the one below that is the best fit for your research. If you are not sure, read the appropriate sections before making your selection.

Life sciences study design

All studies must disclose on these points even when the disclosure is negative.

Sample size	Sample size were selected based on common practices in structural biology and biochemistry
Data exclusions	No exclusion of data were performed in this structural biology study
Replication	All experiments in the manuscript were successfully reproduced
Randomization	Randomization was not required for this structural biology study
Blinding	Blinding was not required for this structural biology study

Reporting for specific materials, systems and methods

We require information from authors about some types of materials, experimental systems and methods used in many studies. Here, indicate whether each material, system or method listed is relevant to your study. If you are not sure if a list item applies to your research, read the appropriate section before selecting a response.

Materials & experimental systems

n/a	Involved in the study	
☒	☐	Antibodies
☒	☐	Eukaryotic cell lines
☒	☐	Palaeontology
☒	☐	Animals and other organisms
☒	☐	Human research participants
☒	☐	Clinical data

Methods

n/a	Involved in the study	
☒	☐	ChIP-seq
☒	☐	Flow cytometry
☒	☐	MRI-based neuroimaging
